# The effect of *Wolbachia* on gene expression in *Drosophila paulistorum* and its implications for symbiont-induced host speciation

**DOI:** 10.1186/s12864-019-5816-9

**Published:** 2019-06-07

**Authors:** Guilherme C. Baião, Daniela I. Schneider, Wolfgang J. Miller, Lisa Klasson

**Affiliations:** 1 Molecular evolution, Department of Cell and Molecular Biology, Uppsala University, Husargatan 3, 751 24 Uppsala, Sweden; 2 Lab Genome Dynamics, Deparment Cell & Developmental Biology, Center of Anatomy and Cell Biology, Medical University of Vienna, Schwarzspanierstraße 17, 1090 Vienna, Austria; 3 Present address: Department of Epidemiology of Microbial Diseases, Yale University, 60 College Street, New Haven, CT 06510 USA

**Keywords:** Speciation, symbiosis, *Wolbachia*, transcriptome, *Drosophila paulistorum*, host-symbiont interactions

## Abstract

**Background:**

The Neotropical fruit fly *Drosophila paulistorum* (Diptera: Drosophilidae) is a species complex *in statu nascendi* comprising six reproductively isolated semispecies, each harboring mutualistic *Wolbachia* strains. Although wild type flies of each semispecies are isolated from the others by both pre- and postmating incompatibilities, mating between semispecies and successful offspring development can be achieved once flies are treated with antibiotics to reduce *Wolbachia* titer. Here we use RNA-seq to study the impact of *Wolbachia* on *D. paulistorum* and investigate the hypothesis that the symbiont may play a role in host speciation. For that goal, we analyze samples of heads and abdomens of both sexes of the Amazonian, Centro American and Orinocan semispecies of *D. paulistorum.*

**Results:**

We identify between 175 and 1192 differentially expressed genes associated with a variety of biological processes that respond either globally or according to tissue, sex or condition in the three semispecies. Some of the functions associated with differentially expressed genes are known to be affected by *Wolbachia* in other species, such as metabolism and immunity, whereas others represent putative novel phenotypes involving muscular functions, pheromone signaling, and visual perception.

**Conclusions:**

Our results show that *Wolbachia* affect a large number of biological functions in *D. paulistorum*, particularly when present in high titer. We suggest that the significant metabolic impact of the infection on the host may cause several of the other putative and observed phenotypes. We also speculate that the observed differential expression of genes associated with chemical communication and reproduction may be associated with the emergence of pre- and postmating barriers between semispecies, which supports a role for *Wolbachia* in the speciation of *D. paulistorum*.

**Electronic supplementary material:**

The online version of this article (10.1186/s12864-019-5816-9) contains supplementary material, which is available to authorized users.

## Background

Speciation is still one of the overarching concepts in biology. The process is usually assumed to involve the development of both pre- and postmating isolation, and a large number of studies have been dedicated to understanding how these arise [1]. One factor which has been gaining attention as a contributor to the speciation process in animals is the influence of microbial symbionts [2]. Growing evidence indicates that microorganisms affect host traits, such as behavior, metabolism, immunity and reproduction, which in turn can influence mating incompatibilities [3]. Insects, in particular, are associated with a large variety of microbial symbionts that are often implicated as contributors to the remarkable species diversity in this group of organisms [2].

The Neotropical fruit fly *Drosophila paulistorum* (Diptera: Drosophilidae) is considered a classical example of incipient speciation. Its six semispecies, Amazonian (AM), Andean-Brazilian (AB), Centro American (CA), Interior (IN), Orinocan (OR) and Transitional (TR) [4, 5] are morphologically similar, have partially overlapping geographical distributions, and yet show both pre- and postmating barriers to hybridization [6, 7]. Premating isolation is observed through female rejection of males belonging to other semispecies [7, 8], while postmating barriers manifest as embryonic lethality and male sterility in the rare hybrids that develop into adults [9, 10]. Early studies suggested that the reproductive incompatibility observed in *D. paulistorum* was due to a microbial infection [11, 12], but it was only recently determined that the microbe in question is *Wolbachia* [8].

*Wolbachia* (Alphaproteobacteria) are widespread endosymbionts of invertebrates, infecting over 60% of insect species [13] as well as Arachnids [14], Crustaceans [15] and Nematodes [16]. They are vertically transmitted through the maternal line and infect primarily the reproductive tissues, although other organs will often also host bacteria [17, 18]. *Wolbachia* have been found to participate in a range of biological interactions with arthropod hosts, from nutritional mutualism and protection against pathogens to various forms of reproductive parasitism [19, 20].

*Wolbachia* have high prevalence among arthropods, but they are often facultative for these hosts. However, in *D. paulistorum, Wolbachia* are obligate mutualists necessary for proper gonad development [8], analogous to what is observed in some wasps of the genus *Asobara* [21, 22]. The mutualistic nature of *Wolbachia* is further supported by its presence in every *D. paulistorum* semispecies tested so far, although the titer of the infection can vary from high to only a few endosymbiont cells per fly [8]. In such low titer cases, *Wolbachia* presence is below the detection limit of a standard PCR, and more sensitive techniques must be used [8]. Remarkably, even very low titer infections are capable of inducing reproductive incompatibility, as successful mating across semispecies is facilitated once the *Wolbachia* titer is reduced through mild antibiotic treatment [8, 11]. Specifically, antibiotic treated females become more accepting of males belonging to other semispecies [8] and hybrid male sterility is partially rescued after treatment of the parents [11]. This suggests that, in this system, the endosymbiont is able to prevent hybridization by inducing not only postmating incompatibility but also premating isolation between semispecies.

Little is known about the influence of *Wolbachia* on biological functions of *D. paulistorum*, but a recent study shows that the symbiont affects male pheromone profiles and thereby modulates mate recognition in that species [23]. This suggests the effect of *Wolbachia* on premating isolation might be associated with changes in host chemical communication [8]. *Wolbachia* has been shown to infect brain regions responsible for sensory perception in *D. paulistorum* [17], and many of the 50 odorant-binding proteins (OBPs) encoded by the *Drosophila* genome could be targets for affecting reception of chemical stimuli [24, 25]. An important group of pheromones in *Drosophila* are the cuticle hydrocarbons (CHCs), molecules derived from fatty acid metabolism [26, 27]. Unique CHC profiles have been associated with each semispecies and sex of *D. paulistorum* [28], and *in vivo* tests demonstrated that cuticular extracts from one semispecies can inhibit courtship by males of others, supporting their role in semispecies isolation [28]. Consequently, *Wolbachia* manipulation of genes related to CHC production and/or perception could affect host premating behavior.

The influence of *Wolbachia* on host postmating compatibility is usually associated with cytoplasmic incompatibility (CI). CI is the most commonly observed *Wolbachia*-induced host manipulation and is characterized by partial or complete embryonic lethality in crosses between infected males and non-infected females or between hosts carrying incompatible symbiont strains. It is not known whether CI has a role in the incompatibilities between *D. paulistorum* semispecies, but the phenotype has been suggested as a driver of speciation in other systems due to its potential to reproductively isolate insect populations [8, 29, 30]. On a cellular level, CI affects paternal chromosome condensation during the first embryonic mitosis, leading to lethal chromatin missegregation in anaphase [31, 32]. Recent studies have also elucidated some of the *Wolbachia* proteins responsible for inducing CI in *D. melanogaster* [31, 33], but very little is known about which host genes are involved in the process.

In the present study, we use RNA-seq to investigate the impact of *Wolbachia* on the biology of three semispecies of *Drosophila paulistorum*, two with low titer *Wolbachia* infections, AM and CA, and one with a high titer *Wolbachia* infection, OR. For each semispecies, we analyze samples from heads and abdomens of both sexes from wild type (WT) flies as well as from corresponding antibiotic-treated and gut flora restored (GFR) individuals. Our goal is to get a better understanding of how *Wolbachia* affects its host and to investigate whether this interaction contributes to the speciation process of *D. paulistorum*. We find that *Wolbachia* affects hundreds of genes associated with global and condition-specific biological processes, including metabolism, immunity, olfactory perception, vision and reproduction. We suggest that the metabolic changes caused by *Wolbachia* might be responsible for other observed phenotypes and discuss the possibility that some of the affected genes and processes support a role for *Wolbachia* in the speciation of *D. paulistorum.*

## Results

### Data, transcriptome assembly and quality assessment

RNA-seq data was collected from heads and abdomens of female and male WT and GFR treated flies of the AM, CA and OR semispecies. While WT flies contain the natural *Wolbachia* titer, GFR flies were subjected to mild antibiotic treatment in order to reduce the *Wolbachia* titer. To avoid host effects stemming from removal of gut microbes, the GFR flies had their gut flora restored after antibiotic treatment (see Materials and Methods). The effectivity of the antibiotic treatment to reduce *Wolbachia* titer in both low and high titer *Wolbachia* in *D. paulistorum* has been previously tested with qPCR [23] (unpublished data). Additionally, we see a strong reduction in the number of reads mapping to *Wolbachia* in samples from GFR compared to WT flies of the high titer semispecies OR (Additional file 1). The absence of differentially expressed (DE) non-*Wolbachia* bacterial genes between WT and GFR samples in all but one condition (data not shown) indicates that gut microbes likely have a very small impact on the results.

Following read quality control, the transcriptomes for the AM, CA and OR semispecies of *D. paulistorum* were assembled separately with Trinity using reads from most samples of that semispecies (see Materials and Methods). Before further analyses, each assembly was then filtered to reduce sequence redundancy and remove non-coding contigs. Additionally, contigs with multiple open reading frames (ORFs) were split. The final reference assemblies showed very high completeness, as measured by recovery of BUSCO markers, and contained between 33000-36000 ORFs each (Table 1). Most ORFs were of *Drosophila* origin, with a minority associated to either bacteria or yeast (Table 1), and the three assemblies show very high overlap in *Drosophila* gene content (Additional file 2: Figure S1). The OR transcriptome was the only one containing *Wolbachia* ORFs (1.43%), which is consistent with OR WT samples having considerably more *Wolbachia* reads than any other condition (Table 1, Additional file 1) and with OR being the only semispecies with a high titer *Wolbachia* infection.

**Table 1:** Assembly metrics for the transcriptomes used in the differential expression analysis of each semispecies

	AM	CA	OR
Number of contigs (ORFs)	35233	36422	33680
Maximum contig length	26253	27348	23729
Average contig length	1384	1284	1374
Contig N50	1923	1781	1889
BUSCO marker recovery (%)			
Arthropod	98.50	98.50	98.78
Insecta	98.01	98.13	98.37
Diptera	95.68	95.61	95.93
ORF completeness (%)			
Complete	41.82	37.32	41.20
3-prime partial	12.74	13.68	13.23
5-prime partial	23.13	23.07	23.41
Internal	22.31	25.93	22.16
ORFs associated to: (%)			
*Drosophila*	77.94	77.28	82.92
*Wolbachia*	0.00	0.00	1.43
Other bacteria	6.36	10.10	3.33
Yeast	1.18	0.98	0.96
Not assigned^a^	14.51	11.64	11.35

^a^ Non-assigned ORFs didn't fulfill our alignment similarity threshold for annotation (see Materials and Methods)

**Fig. 1:**
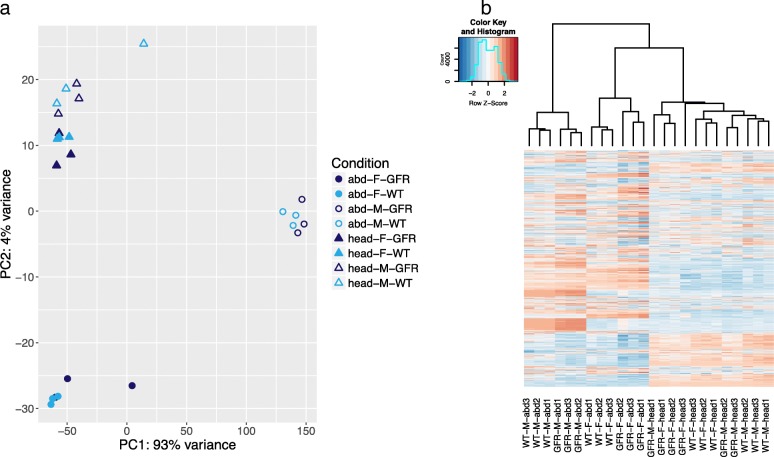
Principal component analysis (**a**) and heatmap (**b**) of expression data of the OR semispecies. The PCA is based on all *Drosophila* genes in the analysis, while the heatmap shows only DE genes. F: female, M: male, WT: wild type, GFR: gut flora restored, abd: abdomen

The reads from each semispecies were mapped to their respective reference transcriptome assembly and counted using FeatureCounts followed by principal component analysis (PCA) in DEseq2. Gene expression in each semispecies varied according to sex, tissue and *Wolbachia* infection, although the latter on a smaller scale (Fig. 1, Additional file 2: Figures S2, S3). Expression differences between sexes were greater in abdomens than in heads, and particularly distinct between male abdomens and other conditions (Fig. 1, Additional file 2: Figures S2, S3). Variation between biological replicates was low (Fig. 1).

### Differential expression analysis in the three semispecies

Separate differential expression analyses were done for each sex and tissue in each semispecies using DESeq2 and an adjusted p-value of 0.05 as significance cutoff. GFR was set as reference condition, which means that the direction of gene expression change is due to *Wolbachia* rather than antibiotic treatment. Since our focus is on investigating the effect of *Wolbachia* on the host gene expression, all results, numbers, figures and discussion presented from here on refer to *Drosophila* genes only, unless otherwise noted.

**Fig. 2:**
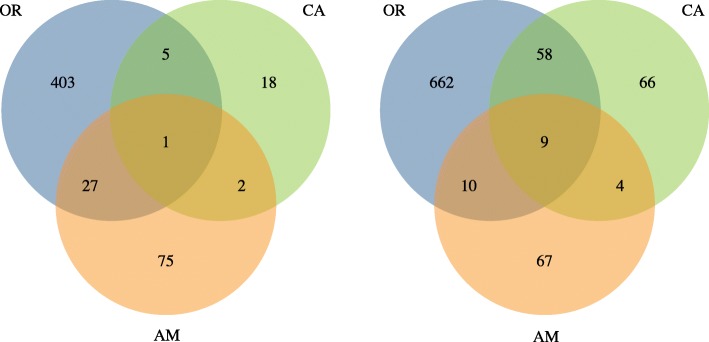
Number of genes up- (**a**) or downregulated (**b**) in one or multiple semispecies. Shared genes are identified as those included in the same OrthoMCL cluster. All conditions are pooled in this analysis and GFR is used as the reference

A total of 175, 209 and 1192 *Drosophila* genes were differentially expressed between WT and GFR flies in AM, CA and OR, respectively. Out of these, 67-81% could be assigned putative functional annotations (Table 2). A heatmap of the DE genes in OR (Fig. 1b) allows visualization of the expression differences between WT and GFR, which clearly are mild compared to differences between tissues and sex. A complete list of DE genes in the three semispecies and their respective annotations is available in Additional files 3, 4 and 5. We identified one up- and 9 downregulated genes which are DE in all three semispecies, irrespectively of condition (Fig. 2). Among these we find *RyR*, a calcium channel which is important for muscle contraction, *FASN2*, which is involved in fatty acid metabolism and implicated in *Drosophila* speciation (see Discussion), the cytochrome P450 gene *Cyp6g1*, and several uncharacterized genes (Additional file 6). On the other hand, a small number of DE genes were present only in one of the assemblies, 4, 2 and 6 genes in AM, CA and OR respectively. None of these were annotated (Additional file 2: Figure S1).

**Table 2:** Number of DE *Drosophila* genes in the three semispecies

Condition	Regulation^a^	AM (annotated)	CA (annotated)	OR (annotated)
Female abdomen	Up	7 (7)	3 (2)	325 (203)
	Down	24 (18)	104 (88)	164 (143)
Female head	Up	59 (50)	18 (14)	36 (23)
	Down	23 (17)	30 (25)	202 (188)
Male abdomen	Up	37 (20)	7 (4)	223 (108)
	Down	7 (5)	15 (14)	324 (282)
Male head	Up	20 (17)	3 (2)	25 (8)
	Down	55 (21)	13 (7)	225 (203)
Total unique DE genes		175 (139)	209 (142)	1192 (921)

^a^ Up- and downregulation presented as a response to *Wolbachia* infection

Most DE genes in the CA and OR semispecies are downregulated in WT (Table 2), and these are proportionally better annotated than upregulated genes in both semispecies. The opposite is seen in AM, where DE genes are more commonly upregulated in WT and these are generally better annotated. (Table 2). In OR, abdomens have more than twice as many DE genes than heads (Table 2). A similar trend is observed in CA, but again the opposite is found in AM (Table 2).

The lower number of DE genes in AM and CA compared to OR (Table 2) might be a consequence of differences in infection titer or *Wolbachia* strains between the semispecies (see Discussion). Given these reduced numbers of DE genes in the AM and CA, from here on we will only present the results from the DE analysis of the OR semispecies, unless otherwise stated.

### Patterns of differential expression in the OR semispecies

**Fig. 3:**
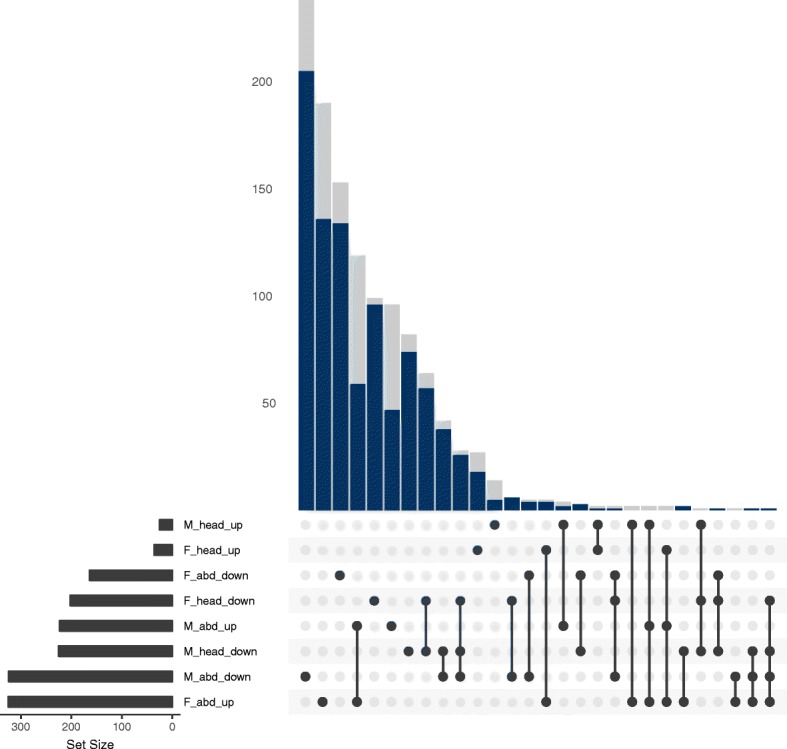
Number of genes differentially expressed in one or multiple conditions of the OR semispecies. A black dot indicates the presence of DE genes for the condition named on the left side. Dots linked by lines represent DE genes in multiple conditions. Vertical bars above the dots correspond to the number of annotated (blue) and unannotated (grey) DE genes present in the condition(s) marked with a dot. Horizontal black bars on the lower left indicate how many genes are DE in each condition. F: female, M: male, WT: wild type, GFR: gut flora restored, abd: abdomen, up: upregulated, down: downregulated

Most DE genes in the three semispecies are DE in a single tissue, sex or condition (i.e. the combination of tissue and sex), with only a small number DE in both sexes or tissues (Fig. 3, Additional file 2: Figures S4, S5). In the OR semispecies, tissue-specific responses include several upregulated genes in both male and female WT abdomens as well as genes downregulated in both male and female WT heads (Fig. 3). Sex-specific responses to *Wolbachia* are less common and more prominent in males, in which 47 genes are downregulated in both abdomens and heads (Fig. 3).

Male abdomens have the largest number of DE genes (Table 2) and female abdomens the highest condition-specificity, with 93% of the down- and 58% of the upregulated genes being exclusive to it (Fig. 3). In heads, males have only a slightly larger number of DE genes than females, but again females have a higher condition-specificity (Table 2, Fig. 3).

### Enrichment of biological process among DE genes in the OR semispecies

Using TopGO and GO term annotation, we analyzed which biological functions were enriched among the DE genes identified in each condition (Table 3, Additional file 7). We found that the DE genes between WT and GFR participate in a wide range of biological processes and are enriched either globally or in specific tissues and conditions, indicating that responses to *Wolbachia* can be either general or localized.

**Table 3:** Ten most significantly enriched GO terms in each condition of the OR semispecies

GO Term	Annotation	Nr Ann.^a^	Nr DE^a^	Nr Exp.^a^	Signif.^a^
**Female abdomen upregulated**					
GO:0045214	sarcomere organization	27	12	0.58	9.20E-14
GO:0030239	myofibril assembly	38	18	0.82	4.90E-09
GO:0007498	mesoderm development	74	12	1.6	4.80E-08
GO:0006936	muscle contraction	23	10	0.5	4.50E-07
GO:0071688	striated muscle myosin thick filament assembly	5	4	0.11	1.00E-06
GO:0007015	actin filament organization	111	12	2.4	2.30E-06
GO:0034446	substrate adhesion-dependent cell spreading	7	4	0.15	7.00E-06
GO:0006941	striated muscle contraction	8	4	0.17	1.40E-05
GO:0060361	flight	10	4	0.22	4.00E-05
GO:0007519	skeletal muscle tissue development	7	3	0.15	0.00033
**Female abdomen downregulated**					
GO:0031122	cytoplasmic microtubule organization	20	5	0.31	9.80E-06
GO:0006270	DNA replication initiation	23	5	0.35	2.10E-05
GO:0007147	female meiosis II	5	3	0.08	3.40E-05
GO:0007338	single fertilization	27	6	0.41	0.00019
GO:0006277	DNA amplification	21	3	0.32	0.00023
GO:0007280	pole cell migration	21	4	0.32	0.00025
GO:0006013	mannose metabolic process	10	3	0.15	0.00038
GO:0071480	cellular response to gamma radiation	10	3	0.15	0.00038
GO:0006517	protein deglycosylation	13	3	0.2	0.00089
GO:0048640	negative regulation of developmental growth	67	5	1.02	0.00131
**Female head upregulated**					
GO:0016059	deactivation of rhodopsin mediated signaling	16	3	0.04	6.60E-06
GO:0042052	rhabdomere development	36	3	0.09	8.10E-05
GO:0007601	visual perception	18	2	0.04	0.00083
GO:0045494	photoreceptor cell maintenance	29	2	0.07	0.00216
GO:0050830	defense response to Gram-positive bacterium	36	2	0.09	0.00331
GO:2000370	positive regulation of clathrin-mediated endocytosis	5	1	0.01	0.01202
GO:0051282	regulation of sequestering of calcium ion	5	1	0.01	0.01202
GO:0051966	regulation of synaptic transmission, glutamatergic	5	1	0.01	0.01202
GO:0050913	sensory perception of bitter taste	5	1	0.01	0.01202
GO:0007604	phototransduction, UV	5	1	0.01	0.01202
**Female head downregulated**					
GO:0002181	cytoplasmic translation	86	28	1.78	9.10E-27
GO:0055114	oxidation-reduction process	472	37	9.78	3.60E-10
GO:0046653	tetrahydrofolate metabolic process	5	4	0.1	8.70E-07
GO:0000028	ribosomal small subunit assembly	12	5	0.25	2.50E-06
GO:0006414	translational elongation	19	5	0.39	3.30E-05
GO:0006730	one-carbon metabolic process	14	4	0.29	0.00015
GO:0006164	purine nucleotide biosynthetic process	83	5	1.72	0.00042
GO:0006635	fatty acid beta-oxidation	31	5	0.64	0.00134
GO:0017085	response to insecticide	12	3	0.25	0.00167
GO:0009620	response to fungus	54	4	1.12	0.00214
**Male abdomen upregulated**					
GO:0045214	sarcomere organization	27	9	0.27	3.00E-12
GO:0030239	myofibril assembly	38	14	0.39	6.50E-09
GO:0014866	skeletal myofibril assembly	7	4	0.07	3.40E-07
GO:0006936	muscle contraction	23	8	0.23	9.80E-07
GO:0060361	flight	10	4	0.1	2.00E-06
GO:0007629	flight behavior	25	5	0.25	4.30E-06
GO:0007498	mesoderm development	74	7	0.75	9.00E-06
GO:0071688	striated muscle myosin thick filament assembly	5	3	0.05	1.00E-05
GO:0007015	actin filament organization	111	8	1.13	1.60E-05
GO:0006099	tricarboxylic acid cycle	34	5	0.35	2.10E-05
**Male abdomen downregulated**					
GO:0032504	multicellular organism reproduction	943	35	23.13	6.50E-16
GO:0055114	oxidation-reduction process	472	37	11.58	5.50E-11
GO:0006508	proteolysis	677	33	16.61	1.50E-06
GO:0009631	cold acclimation	8	4	0.2	2.30E-05
GO:0006629	lipid metabolic process	370	28	9.08	3.30E-05
GO:0042364	water-soluble vitamin biosynthetic process	7	3	0.17	0.00047
GO:0042761	very long-chain fatty acid biosynthetic process	16	4	0.39	0.00051
GO:0045434	negative regulation of female receptivity,postmating	8	3	0.2	0.00074
GO:0006465	signal peptide processing	8	3	0.2	0.00074
GO:0005975	carbohydrate metabolic process	410	22	10.06	0.00187
**Male head upregulated**					
GO:0055093	response to hyperoxia	7	1	0.01	0.0053
GO:0019731	antibacterial humoral response	28	1	0.02	0.0212
GO:0045793	positive regulation of cell size	29	1	0.02	0.0219
GO:0042052	rhabdomere development	36	1	0.03	0.0271
GO:0050830	defense response to Gram-positive bacterium	36	1	0.03	0.0271
GO:0030307	positive regulation of cell growth	40	1	0.03	0.0301
GO:0008286	insulin receptor signaling pathway	42	1	0.03	0.0316
GO:0018105	peptidyl-serine phosphorylation	42	1	0.03	0.0316
GO:0040018	positive regulation of multicellular organism	44	1	0.03	0.0331
GO:0046620	regulation of organ growth	49	1	0.04	0.0368
**Male head downregulated**					
GO:0055114	oxidation-reduction process	472	45	10.14	4.40E-19
GO:1901606	alpha-amino acid catabolic process	35	7	0.75	2.10E-05
GO:0046653	tetrahydrofolate metabolic process	5	3	0.11	9.40E-05
GO:0009620	response to fungus	54	5	1.16	0.00013
GO:0006730	one-carbon metabolic process	14	4	0.3	0.00017
GO:0019236	response to pheromone	15	4	0.32	0.00023
GO:0042559	pteridine-containing compound biosynthetic process	7	3	0.15	0.00032
GO:0072329	monocarboxylic acid catabolic process	40	3	0.86	0.00046
GO:0005977	glycogen metabolic process	15	5	0.32	0.00049
GO:0006098	pentose-phosphate shunt	8	3	0.17	0.0005

^a^*Nr Ann*. Number of times a GO term appears in the reference gene universe. *Nr DE* Number of DE genes which are annotated with the GO term. *Nr Exp.* Number of times a GO term would be expected to appear in the DE genes dataset. *Signif* Significance value in Fishers’ test

In the following sections, we present the main biological functions associated with the DE genes in our dataset based on GO term enrichment, pathway analyses and manual curation (Table 4). For each function, we mention if the response is global or specific and highlight DE genes with high fold change, as these are likely the most reliable and biologically relevant signals in the analysis (Additional files 3, 4 and 5). DE genes with high fold change are those having a fold change higher than at least one standard deviation from the mean fold change of the condition.

**Table 4:** Main biological functions associated with DE genes in the OR semispecies^a^

Gene	Full gene name	F abd	F head	M abd	M head
**Metabolism - Lipids and fatty acids**					
Acsl	Acyl-CoA synthetase long-chain				D
ATPCL	ATP citrate lyase			D	
CDase	Ceramidase			D	
Hmgs	HMG Coenzyme A synthase			D	
Jhe	Juvenile hormone esterase			D	
mag	magro			D	
Sc2	Sc2			D	
bgm	bubblegum			D	D
mino	minotaur			D	D
sPLA2	secretory Phospholipase A2			D	D
Unannotated	22 unannotated genes with homologs in *D. melanogaster*		D/ U	D	D
wal	walrus		D		
yip2	yippee interacting protein 2		D		
FarO	Fatty acyl-CoA reductase in oenocytes	U			
**Metabolism - Purines**					
ade2	adenosine 2		D		
Prat2	Phosphoribosylamidotransferase 2		D		
Unannotated	2 unannotated genes with homologs in *D. melanogaster*		D		
Uro	Urate oxidase	U			
**Metabolism - Amino acids**					
Hn	Henna				D
ppl	pumpless				D
Ssadh	Succinic semialdehyde dehydrogenase				D
Unannotated	6 unannotated genes with homologs in *D. melanogaster*	D	D		D
Ahcy	Adenosylhomocysteinase		D		D
Sardh	Sarcosine dehydrogenase		D		D
Shmt	Serine hydroxymethyl transferase		D		D
Spat	Serine pyruvate aminotransferase		D		D
aay	astray		D		
CG8129	no_fullname		D		
Nmdmc	NAD-dependent methylenetetrahydrofolate dehydrogenase		D		
P5cr-2	Pyrroline-5-carboxylate reductase-like 2		D		
mnd	minidiscs	D			
Gnmt	Glycine N-methyltransferase			D	
Gs2	Glutamine synthetase 2	U			
**Metabolism - Carbohydrates and others**					
Ilp8	Insulin-like peptide 8	D			
LManIII	Lysosomal alpha-mannosidase III	D			
LManVI	Lysosomal alpha-mannosidase VI	D			
AkhR	Adipokinetic hormone receptor				D
Galk	Galactokinase				D
Ilp2	Insulin-like peptide 2				D
Taldo	Transaldolase			D	D
AcCoAS	Acetyl Coenzyme A synthase		D	D	D
Hex-C	Hexokinase C		D	D	D
Idgf6	Imaginal disc growth factor 6		D	D	D
Mdh1	Malate dehydrogenase 1		D	D	D
Unannotated	9 unannotated genes with homologs in *D. melanogaster*		D	D	D
LManII	Lysosomal alpha-mannosidase II		D		D
pug	pugilist		D		D
Pepck	Phosphoenolpyruvate carboxykinase		D		
Amyrel	Amyrel			D	
Cht4	Chitinase 4			D	
Mal-A4	Maltase A4			D	
InR	Insulin-like receptor			U	
LManI	Lysosomal alpha-mannosidase I			U	
Cda5	Chitin deacetylase-like 5	U		U	
kdn	knockdown	U		U	
rgn	regeneration	U		U	
Idgf4	Imaginal disc growth factor 4	U			
boss	bride of sevenless		U		
**Proteolysis**					
LUBEL	Linear Ubiquitin E3 ligase	D		D	
26-29-p	26-29kD-proteinase			D	
Ance	Angiotensin converting enzyme			D	
cathD	cathD			D	
iotaTry	iotaTrypsin			D	
CtsB1	Cathepsin B1			D	D
Acer	Angiotensin-converting enzyme-related		D		D
Ance-5	Ance-5		D		D
Unannotated	30 unannotated genes with homologs in *D. melanogaster*	D/ U	D	D	D
gol	goliath		D		
Jon99Ci	Jonah 99Ci	D	D		
Ance-3	Ance-3	D			
Bace	beta-site APP-cleaving enzyme	D			
CG13025	no_fullname	U			
CG2224	no_fullname	U			
dmpd	dampened	U			
e(y)2b	enhancer of yellow 2b	U			
Jon65Aiv	Jonah 65Aiv	U			
Jon66Cii	Jonah 66Cii	U			
Npl4	Nuclear protein localization 4	U			
SP1029	SP1029	U			
Usp30	Ubiquitin specific protease 30	U			
epsilonTry	epsilonTrypsin	U		U	
Prosalpha4	Proteasome alpha4 subunit			U	
**Immunity**					
Rel	Relish		D		
Glt	Glutactin		D		D
GNBP-like3	GNBP-like 3		D		D
MP1	Melanization Protease 1		D		D
GNBP2	Gram-negative bacteria binding protein 2				D
Tsf1	Transferrin 1				D
SPE	Spatzle-Processing Enzyme			D	D
Spn28Dc	Serpin 28Dc			D	D
yellow-f2	yellow-f2		D	D	D
casp	caspar			D	
Hsp27	Heat shock protein 27			D	
PPO2	Prophenoloxidase 2			D	
CBP	sarcoplasmic calcium-binding protein	D			
Dlip3	Dorsal interacting protein 3	D			
Hat1	Histone acetyltransferase 1	D			
heix	heixuedian	D			
Unannotated	4 unannotated genes with homologs in *D. melanogaster*	U	D	U/ D	D
AttA	Attacin-A				U
LysD	Lysozyme D			U	
Lmpt	Limpet	U		U	
AttD	Attacin-D	U	U		
DptB	Diptericin B	U	U		
AttC	Attacin-C	U			
e	ebony	U			
edin	elevated during infection	U			
Fer2LCH	Ferritin 2 light chain homologue	U			
Fuca	alpha-L-fucosidase	U			
pirk	poor Imd response upon knock-in	U			
Tep3	Thioester-containing protein 3	U			
Tep4	Thioester-containing protein 4	U			
yellow-b	yellow-b	U			
**Perception - Olfactory**					
Est-6	Esterase 6		D		D
Obp56a	Odorant-binding protein 56a		D		D
Obp99c	Odorant-binding protein 99c		D		D
Obp56g	Odorant-binding protein 56g				D
Obp56h	Odorant-binding protein 56h				D
Obp83ef	Odorant-binding protein 83ef				D
Obp99b	Odorant-binding protein 99b			D	D
Obp56d	Odorant-binding protein 56d	U		D	D
**Perception - Vision**					
ninaD	neither inactivation nor afterpotential D				D
chp	chaoptin		U		
eys	eyes shut		U		
Fbxl4	F box and leucine-rich-repeat gene 4		U		
ninaC	neither inactivation nor afterpotential C		U		
ninaG	neither inactivation nor afterpotential G		U		
norpA	no receptor potential A		U		
prom	prominin		U		
**Pheromone production**					
Desat2	Desaturase 2			D	
FASN1	Fatty acid synthase 1			D	
FASN2	Fatty acid synthase 2			D	
Fbp2	Fat body protein 2			D	
Fbp1	Fat body protein 1		D	D	
**Reproduction - Females: Cell cycle, germline development**					
alphaTub67C	alpha-Tubulin at 67C	D			
aPKC	atypical protein kinase C	D			
BicC	Bicaudal C	D			
cad	caudal	D			
CDC45L	CDC45L	D			
cort	cortex	D			
CycB3	Cyclin B3	D			
dhd	deadhead	D			
egg	eggless	D			
exu	exuperantia	D			
fs(1)Ya	female sterile (1) Young arrest	D			
Fs(2)Ket	Female sterile (2) Ketel	D			
fzy	fizzy	D			
gcl	germ cell-less	D			
Grip84	Gamma-tubulin ring protein 84	D			
gus	gustavus	D			
hop	hopscotch	D			
lok	loki	D			
Mcm10	Minichromosome maintenance 10	D			
Mer	Merlin	D			
Mos	Mos oncogene	D			
nos	nanos	D			
pbl	pebble	D			
Pen	Pendulin	D			
pie	pineapple eye	D			
png	pan gu	D			
polo	polo	D			
slam	slow as molasses	D			
spd-2	spindle defective 2	D			
ssh	slingshot	D			
stai	stathmin	D			
swa	swallow	D			
tor	torso	D			
Tre1	Trapped in endoderm 1	D			
twe	twine	D			
Xpc	Xeroderma pigmentosum%2C complementation group C	D			
asp	abnormal spindle		D		
cmet	CENP-meta		D		
spn-E	spindle E		D		
Mdr49	Multi drug resistance 49			D	
Rab1	Rab1			D	
kug	kugelei			U	
Doa	Darkener of apricot	U		U	
bond	james bond	U			
c(3)G	crossover suppressor on 3 of Gowen	U			
LanA	Laminin A	U			
**Reproduction - Males: Regulation of postmating behavior**					
antr	antares			D	
aqrs	aquarius			D	
CHOp24	CHOp24			D	
Esp	Epidermal stripes and patches			D	
to	takeout			D	
EbpIII	Ejaculatory bulb protein III	U	D	D	D
**Muscular functions**					
Fkbp14	FK506-binding protein 14				D
Mical	Molecule interacting with CasL			U	
SERCA	Sarco/endoplasmic reticulum Ca(2+)-ATPase			U	
sesB	stress-sensitive B			U	
skd	skuld			U	
Actn	alpha actinin	U		U	
bt	bent	U		U	
clumsy	Clumsy	U		U	
fln	flightin	U		U	
Mhc	Myosin heavy chain	U		U	
Mhcl	Myosin heavy chain-like	U		U	
Mlc1	Myosin alkali light chain 1	U		U	
Mlc2	Myosin light chain 2	U		U	
Mlp60A	Muscle LIM protein at 60A	U		U	
Msp300	Muscle-specific protein 300 kDa	U		U	
shot	short stop	U		U	
Tm2	Tropomyosin 2	U		U	
tn	thin	U		U	
TpnC4	Troponin C isoform 4	U		U	
uif	uninflatable	U		U	
Unannotated	28 unannotated genes with homologs in *D. melanogaster*	U		U	
Unc-89	Unc-89	U		U	
up	upheld	U		U	
vkg	viking	U		U	
Zasp52	Z band alternatively spliced PDZ-motif protein 52	U		U	
Alk	Anaplastic lymphoma kinase	U			
CAP	CAP	U			
chas	chascon	U			
Col4a1	Collagen type IV alpha 1	U			
eya	eyes absent	U			
Grip	Glutamate receptor binding protein	U			
if	inflated	U			
Mlp84B	Muscle LIM protein at 84B	U			
Neurochondrin	Neurochondrin	U			
RyR	Ryanodine receptor	U			
sals	sarcomere length short	U			
TpnC73F	Troponin C at 73F	U			
Zasp66	Z band alternatively spliced PDZ-motif protein 66	U			
**Translation**					
Tfb4	Transcription factor B4	D			
eEF1gamma	eukaryotic translation elongation factor 1 gamma		D		
eEF2	eukaryotic translation elongation factor 2		D		
eEF5	eukaryotic translation elongation factor 5		D		
Rack1	Receptor of activated protein kinase C 1		D		
sta	stubarista		D		
eEF1alpha1	eukaryotic translation elongation factor 1 alpha 1		D		D
RpL/ RpS	28 Ribosomal proteins		D	D	D
eEF1alpha2	eukaryotic translation elongation factor 1 alpha 2	U		U	
**Cytochrome P450**					
Cyp311a1	Cyp311a1			D	
Cyp4e2	Cytochrome P450-4e2			D	
Cyp6a8	Cytochrome P450-6a8			D	
Cyp6d2	Cyp6d2			D	
Cyp12a4	Cyp12a4		D		
Cyp12d1-p	Cyp12d1-p		D		
Cyp6t1	Cyp6t1		D		
Cyp309a2	Cyp309a2		D		D
Cyp6g1	Cyp6g1		D		D
Cyp4p3	Cyp4p3		D	D	D
Cyp6d5	Cyp6d5		D	D	D
Cyp4ac2	Cyp4ac2				D
Cyp4e3	Cytochrome P450-4e3			U	
**Yolk proteins**					
Yp1	Yolk protein 1				D
Yp2	Yolk protein 2				D
Yp3	Yolk protein 3				D

^a^ *F* Female, *M* Male, *Abd* abdomen, *D* Downregulated in WT, *U* Upregulated in WT

### Metabolism

A large number of genes involved in carbohydrate, fatty acid and amino acid metabolism are DE in multiple conditions, often with high fold changes (Additional file 3). Most of these are downregulated in WT flies*,* particularly in female and male heads and in the male abdomens (Table 4).

**Fig. 4:**
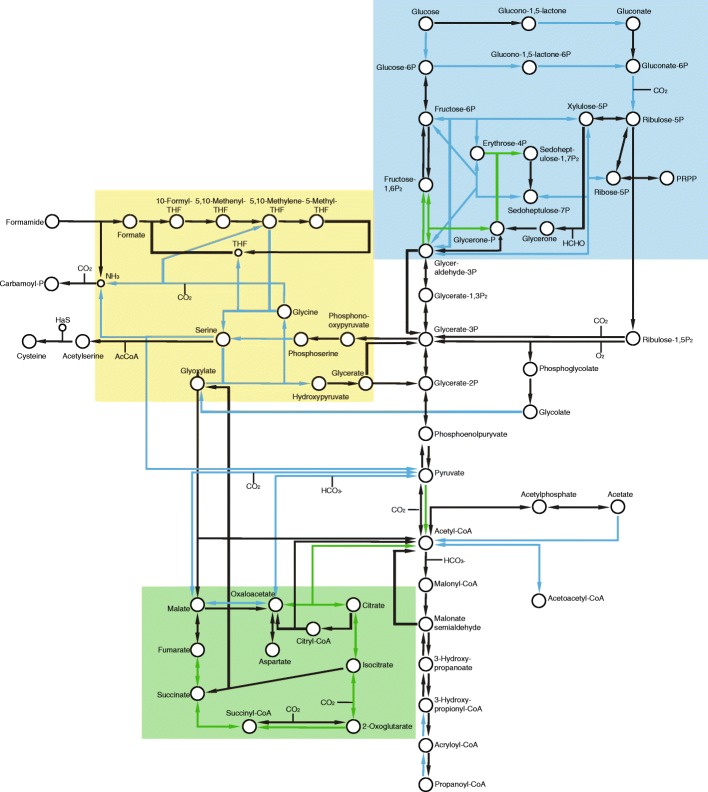
Metabolic map of differentially expressed genes in carbon and energy metabolism of *Drosophila paulistorum.* Upregulated genes are shown in green and downregulated genes in blue. DE genes from all conditions of the OR semispecies are represented. Green box: TCA cycle. Yellow box: Glycine and serine metabolism. Blue box: pentose phosphate pathway. The figure is redrawn based on KEGG map01200

The precise identities of the affected genes differ somewhat between conditions, even so, a relatively clear pattern exists for up- and down-regulated genes in glucose and energy metabolism (Fig. 4). In WT flies, there is an increased expression of genes involved in the TCA cycle (abdomens), whereas in GFR flies there is an increased expression of genes involved in the pentose phosphate pathway, the breakdown of glycogen to UDP-glucose (males) and beta-oxidation of fatty acids (female heads). This pattern suggests increased catabolism in WT flies, possibly with an increased flux through the TCA cycle, whereas GFR flies have more anabolic metabolism and use beta-oxidation for producing acetyl-CoA as well as the pentose phosphate pathway for generating precursors for nucleotide, amino acid or lipid biosynthesis.

#### Lipids

The putative increased anabolism in GFR flies is supported by the upregulation of genes involved in fatty acid biosynthesis and phospholipid metabolism in multiple conditions (Table 4). These include several genes in pathways for converting glycerate to phosphatidic acid, phosphatic acid to phosphatidylethanolamine and phosphatidylcholine, and ethanolamine to phosphoethanolamine.

Furthermore, GFR flies have a higher expression of five genes putatively involved in cholesterol hydrolysis (*CG8093*, *CG31091*, *CG2772*, *CG7329* and *CG18302*) as well as three genes containing the Niemann-Pick type C-2 domain, which has a potential role in intracellular cholesterol transport.

#### Nucleotides

Several genes related to purine biosynthesis are upregulated in the head of female GFR flies, which further supports their increased anabolism. The presence of genes for *de novo* purine synthesis in *Wolbachia* genomes indicates that the symbiont is likely able to synthesize such molecules, and the increased expression of purine biosynthesis genes in GFR flies might thus be a compensation for the loss of *Wolbachia*-provided purines. In filarial nematodes, where *Wolbachia* is also an obligate mutualist, it has been suggested that one of its functions is to provide purines for the host [34].

#### Amino acids

Although *Wolbachia* relies on the host for obtaining most amino acids [35], we only observe one amino acid biosynthesis gene with a higher expression in WT flies (*glutamine synthetase 2*). Hence, increased host biosynthesis is likely not a source of amino acids for *Wolbachia*. However, and again in agreement with the putative increased anabolism in GFR flies, some amino acid biosynthetic genes have lower expression in WT flies. These include two genes in the pathway converting glutamate to proline and several genes involved in serine and glycine metabolism (Table 4, Fig. 4), all with lower expression in male and/or female heads. Collectively, this suggests that both glycine and serine levels might be reduced in the heads of WT flies.

Furthermore, two putative components of the glycine cleavage system are downregulated in heads of WT males and females. This is a further indication that the level of glycine is lower in WT flies, as the activity of this system is normally regulated by the concentration of glycine*.* Apart from its role in glycine breakdown, the glycine cleavage system uses tetrahydrofolate (THF) to generate 5,10-Methylene-THF, which can be further utilized in purine biosynthesis or as a substrate for the enzyme serine hydroxymethyl transferase. Several additional genes involved in THF conversions, such as *pug* and *CG34424*, or in purine biosynthesis, like *Nmdmc* and *CG11089*, also have a lower expression in heads of WT flies. An increase in THF conversion is a further indication of anabolic metabolism in GFR flies, as these conversions are mainly performed by enzymes involved in biosynthesis of amino acids and nucleotides.

We note that most of the above-mentioned expression changes manifest in heads and that glycine acts as a neurotransmitter which has both serine and proline as agonists. Several putative transporters of glycine and proline are encoded in *Wolbachia* genomes from various *Drosophila* species, and among the few genes for amino acid synthesis found in these genomes are those that can perform serine to glycine and threonine to glycine conversions.

#### Metabolic hormones

Both *insulin-like peptide 2* and *8* have lower expression in WT flies, and the *adipokinetic hormone* (insect glucagon) *receptor* (*AkhR*) has significantly lower expression in heads of male WT flies. Also the G-couple receptor encoded by *boss* has increased expression in heads of WT female flies. *Boss* is involved in regulation of sugar and lipid metabolism, and loss of function mutants show symptoms that resemble those of flies with defective insulin signaling [36]. Once again, this suggests that GFR flies have more nutrients available and a predominantly anabolic metabolism whereas WT flies have reduced nutrient availability and more catabolic metabolism.

### Proteolysis

Although the GO term proteolysis is only enriched in downregulated genes in male abdomens, genes containing protease or peptidase domains are DE in various conditions, sometimes with high fold change. These DE genes are involved in various biological functions, but since the majority are downregulated in WT flies, the overall breakdown of proteins and consequent release of free amino acids appears to be lower in WT flies. One of the DE genes associated with proteolysis is a component of the proteasome (*Proteasome alpha4 subunit*). Proteins destined for degradation by the proteasome are tagged with ubiquitin, and a few genes involved in ubiquitination and deubiquitination, such as *LUBEL*, are also DE (Table 4).

Four different serpins (serine protease inhibitors) are downregulated in male abdomens, some of which are also DE in other conditions (Additional file 3). If these serpins inhibit proteases, this pattern indicates increased proteolysis in the presence of higher *Wolbachia* titer. However, two of them (*Spn43Ab* and *Spn75F*) are classified as non-inhibitory serpins and may have roles in reproduction, with *Spn75F* being produced by the male accessory gland [37]. Of the remaining two, *Spn28Dc* inhibits spontaneous melanization and is necessary for pupal viability, while *Spn42Da* might be involved in retention of proteins in the endoplasmic reticulum [37].

### Immunity

The effect of *Wolbachia* on immunity genes is a global response in the host, being observed in both sexes and tissues (Table 4).

One of the most important constituents of the insect immune system are the antimicrobial peptides (AMPs), small proteins which are active against a variety of bacteria, protozoans, fungi and viruses [38]. Several AMPs are upregulated with high fold changes in WT flies (Additional file 3), including three attacins and *Diptericin B*. *Attacin A* and *Diptericin B* are also upregulated in WT males of the AM semispecies (Additional file 5).

*Wolbachia* also affects the expression of several genes involved in the Toll and IMD pathways, which regulate AMP induction. DE components of the Toll pathway include two gram negative binding proteins (*GNBP2* and *GNBP-like3)* and the Spatzle processing enzyme (*SPE*), all of which are downregulated in various conditions of WT flies (Table 4). *GNBP-like3* is also downregulated in AM (female heads) and CA (female abdomens) flies, while the peptidoglycan recognition proteins *PGRP-SD* and *PGRP-SB1* are upregulated in AM WT flies (male heads and abdomens) (Additional files 4 and 5). Several regulators of the IMD pathway are also present among the DE genes and include *Relish,* which modulates expression of diptericins and attacins [39], *heix*, and the negative regulators *caspar* and *SC2,* all of which are downregulated in WT flies.

A number of DE genes are also associated with melanization, another important innate immune response in *Drosophila*. This is the case for Prophenoloxidase, *MP1 and Yellow-f2*, the latter is also DE in the CA semispecies (Table 4, Additional file 4). The immune gene *edin,* whose expression in the fat body is induced by bacterial infections [40], is upregulated with high fold change in OR WT flies and in males of WT AM flies (Table 4, Additional files 3, 4 and 5). Additionally, we identify DE genes linked to regulation of iron availability (*Tsf1, Fer2LCH*), response to fungus (*Lmpt, CG9372)* and opsonization (*Tep3*, *Tep4*) (Table 4).

### Perception

Several genes associated with sensory perception are DE in multiple conditions, suggesting *Wolbachia* affects how *D. paulistorum* perceives its environment. This global response includes several odorant-binding proteins which are associated with the enriched GO terms “Response to pheromone”, and “sensory perception of smell” (Table 3, Additional file 7). Most DE OBPs are downregulated in WT male heads (Table 4), a few of which with high fold change (Additional file 3). Additionally, Esterase 6*,* which affects the speed of odorant recognition [41], is downregulated in heads of WT flies of both sexes.

We also find several genes related to visual perception and eye development upregulated in female heads (Table 4), among which *chaoptin*, *eyes shut* and *ninaG* are DE with high fold changes (Additional file 3)*.* Several GO terms associated with visual functions are enriched in the same condition (Table 3).

### Pheromone production

Since cuticle hydrocarbon production is dependent on fatty acid metabolism, DE genes associated with such processes are candidates for affecting pheromone production. Among these, *FASN1, FASN2* and *desat2*, all of which are downregulated in WT flies, have previously been implicated in *Drosophila* speciation (see Discussion). Other genes associated with fatty acid metabolism but so far with no described influence on pheromone synthesis are *Bubblegum,* which mediates activation of long chain fatty acids for synthesis and degradation of cellular lipids [42] and *FarO*, a fatty acyl-CoA reductase with activity in oenocytes, the cells which produce CHCs (Table 4). Also the fat body proteins *Fbp1* and *Fbp2,* which participate in import of storage proteins into the fat body [43], might be involved in transport of pheromone precursors and are downregulated with high fold change in WT flies (Table 4, Additional file 3). *Fbp1* is also DE in AM female abdomens (Additional file 5).

### Reproduction

Many GO terms related to reproduction are enriched among downregulated genes in both male and female abdomens of WT flies (Table 3). In females, these involve several replication and cell-cycle-associated functions, as well as female-specific reproductive processes such as oogenesis, oocyte maturation, egg activation and germ cell migration. The DE genes in female abdomens associated with reproduction contribute to the high condition-specificity observed in this condition. In males, the enriched GO term with the largest number of reproduction-related genes is the broad “multicellular organism reproduction”. Most genes in this category encode proteins of unknown functions that are inferred to be involved in reproduction through indirect evidence, for example by the fact that they are specifically expressed in the male accessory gland of *D. melanogaster*.

#### Females

Among the many reproduction-associated genes downregulated by *Wolbachia* in WT female abdomens are the two meiotic regulators *Twine* and *Polo*. Both are involved in activating Cyclin-dependent kinase 1 (*CDK1*)/*Cyclin B*, which in turn is required for releasing the oocyte from the prophase I primary meiosis arrest [44, 45]. *CyclinB3*, which has been shown to be involved in oocyte maturation, is downregulated. Furthermore, *Cortex* and *Fizzy*, two activators of the Anaphase promoting complex (APC/C) which is necessary for metaphase to anaphase transition, are downregulated in WT flies.

As a further indication of *Wolbachia*’s effect on cell cycle, several genes involved in spindle formation and microtubule dynamics are also downregulated in WT flies. These include two subunits of the augmin complex that is involved in microtubule-dependent nucleation by recruitment of gamma-tubulin to the spindle (Table 4). Furthermore, *Pan gu*, implicated in the translational control of a large majority of mRNAs during egg activation, also has a lower expression in WT female abdomens, although the two activating proteins that usually form a complex with it are not affected.

Finally, since the first cell divisions after fertilization of the egg are controlled by maternal mRNAs and proteins, early embryonic development can also be affected by female DE genes. Several such genes are downregulated in WT female abdomens and include *Deadhead*, which is involved in male pronucleus activation after sperm entry into the egg, *female sterile (1)* and *Young arrest*, which is necessary for mitotic phase initiation during early embryogenesis. Additionally, many of the maternal effect genes involved in defining anterior-posterior polarity of the egg and embryo have lower expression in WT female abdomens. *Nanos* and *caudal*, two of the critical components for regulation of the posterior part of the embryo, as well as *exuperantia* and *swallow*, both of which interact with the anterior localization of *bicoid* mRNA, all have a lower expression in WT female abdomens (Table 4). Several other genes important for the development and migration of germ cells are also downregulated by *Wolbachia*, such as *germ cell-less* (*gcl*), whose low expression causes females to produce sterile offspring without germ cells [46], and *gustavus* (*gus*), involved in localizing *vasa* to the posterior end of the embryo and needed for primordial germ cell development [47].

#### Males

Several reproduction-related genes are DE in males, but only a handful have known functions. As in females, these genes are all downregulated in WT flies. Among the genes with known functions are several associated with post mating modulation of female receptivity and egg production. The two proteins *aquarius* and *antares*, for example, are necessary to facilitate the bond between sperm and sex peptide, a seminal protein known to increase production of eggs and decrease receptivity in mated females. Knocking down the expression of these genes in *D. melanogaster* males result in disturbed release of sperm from storage and reduced long term fertility in mated females [48].

The Angiotensin converting enzyme, *Ance*, suggested to have a role in spermatogenesis, has previously been considered a possible CI target [49]. *Ance* is downregulated in male WT abdomens while *Ance-3* is upregulated in female WT abdomens, which supports the previous *in vivo* observation that *Ance* expression is higher in infected ovaries but lower in infected testes of *D. simulans* and *D. melanogaster* [49]. The genes *Ance-5* and *Acer* (Angiotensin-converting enzyme-related), however, are here downregulated in the heads of WT flies of both sexes.

Two genes that affect male mating behavior are also downregulated in WT male abdomens. These are *Takeout*, a sex specific factor shown to influence courtship behavior in a non-pheromone dependent way [50] and Juvenile hormone esterase.

Finally, the Ejaculatory bulb protein III, a protein component of the posterior mating plug, is differentially expressed not only in male abdomens but in all sexes and tissues (Table 4).

### Muscular functions

We find a large number of upregulated genes associated with enriched muscle-related GO terms in both male and female WT abdomens (Tables 3 and 4). Most of these encode structural components of the sarcomere, the basic unit of skeletal and cardiac striated muscles (Table 4), and many are DE with high fold change (Additional file 3). Other upregulated genes include the ryanodine receptor (*RyR*), which appears DE in all three semispecies and is involved in calcium channeling, and the sarcoendoplasmic reticulum Ca^2+^ ATPase (*SERCA*), involved in muscle contraction (Table 4). The fact that *Drosophila* ventral abdominal muscles are innervated by glutamatergic synapses might also be the reason why genes associated with glutamate metabolism and reception such as *Grip*, *Gs2* and *clumsy*, are upregulated in abdomens (Table 4). Taken together, these results indicate that *Wolbachia* is either directly or indirectly affecting muscle contraction.

### Translation

A large number of translation-associated genes are downregulated in heads of both sexes, albeit in higher numbers in females where translation is also an enriched GO term (Table 3). In total, 36 ribosomal proteins and four elongation factors are downregulated by *Wolbachia* (Table 4) in WT flies, suggesting that *Wolbachia* reduces host translation at least in female heads.

### Cytochrome P450

Cytochrome P450 is a family of heme-containing proteins which in *D. melanogaster* is associated with detoxification, production of the hormone 20-hydroxyecdysone and various behavioral and reproductive phenotypes [51]. In *D. paulistorum*, *Wolbachia* downregulates several cytochrome P450 genes, most of which are poorly characterized (Table 4). Studies with their orthologs in *D. melanogaster* suggest that *Cyp311a1* is essential for larval development and that *Cyp12d1-p*, *Cyp6g1*, *Cyp4s3*, *Cyp6a8* and *Cyp12a4* have a role in detoxification, while defects in either *Cyp4ac2* or *Cyp4s3* lead to lower fitness. [51].

### Yolk proteins

Three yolk proteins (*Yp1*, *Yp2, Yp3*) are downregulated in WT male heads, two of them with high fold change (*Yp1*, *Yp3*) (Additional file 3). The gene *CG5966*, downregulated with high fold change in WT female heads, has the same Lipase/Vitellogenin domains found in yolk proteins, which suggests it may be involved in similar responses in the female head.

### Does *Wolbachia* contribute to differences in semispecies-specific gene expression?

In order to investigate if *Wolbachia* could contribute to speciation via changes in gene expression between semispecies, we mapped reads from all semispecies to the same transcriptome. The three assemblies show very high overlap with each other (Additional file 2: Figure S1), but since most of our analyses are focused on OR, we selected this transcriptome as reference. This choice is supported by the fact that the number of mismatches per base and the percentage of mapped reads obtained when mapping AM and CA to OR are similar to those seen when those semispecies are mapped to their own references (Additional file 1).

**Fig. 5:**
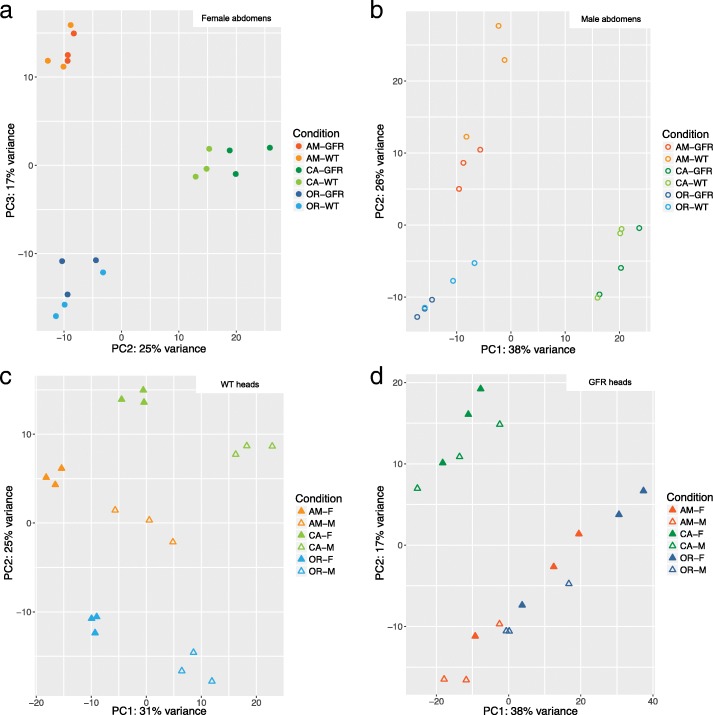
Principal component analysis of all semispecies mapped to the OR transcriptome. (**a**): Female abdomens, (**b**): Male abdomens, (**c**): WT male and female heads, (**d**): GFR male and female heads. F: female, M: male, WT: wild type, GFR: gut flora restored, abd: abdomen

The PCA produced from these mappings showed that the three semispecies could be discriminated by their gene expression in both sexes and tissues (Fig. 5). The first and second principal components (PCs) separated the semispecies in all conditions except female abdomens, in which they were distinguished by PC2 and PC3 (Fig. 5, Additional file 2: Figure S6). We could not identify any particular factor associated with PC1 in the female abdomen.

The three semispecies were clearly separated in a PCA of the WT head samples, but were less clearly separated in the corresponding GFR plot (Fig. 5c, d, Additional file 2: Figures S7, S8). Additionally, we found that DE genes between WT and GFR head samples in the OR and AM semispecies are significantly overrepresented (Chi^2^ test, p < 0.01) among the genes that contribute the most to separating the semispecies in the PCA of WT head samples. Taken together, our results indicate that *Wolbachia* might contribute to the difference in expression pattern between heads of the three abdomens, although sex (PC1) explains 98% of the separation between the WT samples, a distinction between semispecies is seen when PC2 and PC3 are plotted (Additional file 2: Figure S9). Similar to what we detected in heads, DE genes between WT and GFR abdomen samples in the OR semispecies are overrepresented (Chi^2^ test, p < 0.01) among the genes that contribute most to the semispecies separation. This suggests that *Wolbachia* may also contribute to gene expression differences between abdomens of the three semispecies, although in a more subtle way than in heads.

## Discussion

Differential expression analysis between WT and GFR flies of the AM, CA and OR *D. paulistorum* semispecies revealed *Wolbachia*-induced changes on a wide range of host biological processes, particularly in the OR semispecies. Some of the most prominent effects are seen in metabolism, reproduction, immunity and muscular functions. Several differentially expressed genes possibly involved with production and reception of pheromones may have implications for host mating behavior and speciation.

The strategy of performing separate *de novo* transcriptome assemblies for AM, CA and OR was chosen in order to preserve potentially unique contigs of each semispecies. The relatively high percentage of ORFs which remained unannotated after similarity searches against both *D. willistoni* and *D. melanogaster* (Table 1) is likely an indication of sequence divergence between these species and *D. paulistorum*. This suggests *de novo* assembly as not only a suitable but potentially necessary approach. A high number of unannotated DE genes was found in both our analysis and in other systems [49, 52, 53], further showing the need for a *de novo* approach and also clearly demonstrating our incomplete understanding of the biology of *Wolbachia*–*Drosophila* interactions.

The use of different reference assemblies for the three semi-species also allowed us to detect a few DE genes that are specific to a semispecies. The percentage of reads mapping back to the assembled transcriptomes, the proportion of complete ORFs and the number of BUSCO marker genes recovered indicate that the assemblies of all three semispecies are of high quality and completeness (Table 1).

### Influence of experimental setup and *Wolbachia* strain on differential expression results

Although other studies have investigated the influence of *Wolbachia* on gene expression in insect hosts [49, 54, 55], none has analyzed the influence across different tissues and sexes separately. Using whole flies or cell lines might dilute the signal or blur the specificity of the biological response which, as we see from our results, is often tissue specific. Thus, using multiple tissues and both sexes is expected to be a more precise strategy for transcriptome studies between *Wolbachia* and host. As a likely result of this method, the 1192 DE genes found in OR (Table 2) are considerably more than the 250-450 DE genes reported in a number of previous studies [49, 52–54].

On the other hand, the numbers of DE genes for AM and CA (Table 2) are in line with other studies of *Wolbachia* and host gene expression, and consequently lower than those in OR. A likely explanation for the smaller number of DE genes in AM and CA is the low *Wolbachia* infection titer in these two semispecies compared to OR [8]. This difference in *Wolbachia* titer is illustrated by the number of reads and ORFs associated with *Wolbachia* in each semispecies (Table 1, Additional file 1) but is also known from previous studies [8, 23]. In the lower titer infections, it is possible that extracting RNA from whole heads and abdomens might have diluted the signal, since probably only a small number of cells are infected with *Wolbachia*. Hence, analyzing infected and uninfected cells separately might thus be necessary in order to detect differential expression in low titer infections. Alternatively, it is also possible that the observed differences between AM, CA and OR are a consequence of the three semispecies being infected with different *Wolbachia* strains. Previous studies have shown that which host genes are differentially expressed in response to *Wolbachia* can differ according to the infecting *Wolbachia* strain [52]. Unfortunately, we currently do not know what the genetic differences between the strains are, and are therefore unable to test or speculate further about this.

Given that we don’t fully understand the biological reason behind the observed differences between the semispecies, we can’t completely discard the hypothesis that the lower numbers of DE genes in AM and CA also mean a reduced or divergent impact of the symbiont on these hosts compared to OR. However, we notice that several functional categories are enriched in all the semispecies (Table 3, Additional file 7). This fact not only supports previous observations that low titer AM and CA *Wolbachia* have a relevant impact on host biology [8, 11, 12, 23], but also suggest similarities on host effects in the three semispecies. In AM, this is seen as upregulated muscle functions in male abdomens, upregulated visual function in the female heads, upregulated defense genes in male and female heads and downregulated metabolic processes in female heads (Additional file 7). In CA, we observe upregulation of immunity genes in male abdomens and downregulation of carbohydrate metabolism in female abdomens (Additional file 7). Interestingly, even though these functional categories are affected in multiple semispecies, only a small overlap exits in the actual DE genes (Fig. 2). This again suggests a relatively high specificity in the interactions between each *Wolbachia* and its *D. paulistorum* host.

In the next sessions, we discuss the main biological functions affected by *Wolbachia* in the *D. paulistorum* host. Both previously known and novel/putative functions are discussed and, whenever relevant, we consider how the affected genes might support the hypothesis that *Wolbachia* contributes to host speciation.

### Functions previously known to be affected by *Wolbachia*

#### Metabolism

Given its obligate intracellular lifestyle and small genome with limited gene content, it is clear that *Wolbachia* is not able to produce all the nutrients it needs and thus must obtain them from the host. As a likely consequence of this, we find that many genes involved in metabolic and biosynthetic processes are DE between WT and GFR flies. High *Wolbachia* levels are associated with upregulation of genes in the TCA cycle and a generally more catabolic metabolism. Low *Wolbachia* levels, on the other hand, have increased expression of genes involved in beta-oxidation and the pentose phosphate pathway, an indication of anabolic metabolism and active production of precursors for nucleotide, amino acid and lipid biosynthesis. These differences in gene expression are similar to those observed in protein expression of *D. melanogaster* on a poor vs. a rich diet [56], suggesting that being infected with *Wolbachia* may have a significant metabolic cost for the host.

In contrast to what we described for genes in glucose and energy metabolism, several of the downregulated genes associated with amino acid metabolism were recently shown to have a higher expression in starved compared to non-starved brains of *D. melanogaster* [57]. The upregulation of these genes is correlated with high serine levels and starvation-induced sleep suppression [57]. Hence, the expression pattern of the serine and glycine metabolism genes in heads of GFR flies, rather than WT flies, mimic starvation conditions. Interestingly, in line with this observation, a recent study showed an increase in nighttime activities in non-*Wolbachia* infected *D. melanogaster* flies compared to infected [58].

Several studies have shown that *Wolbachia* rely on cholesterol from the host, a property which is also believed to be important for the pathogen blocking phenotype of the symbiont [59, 60]. We observe that many genes involved in fatty acid and lipid metabolism are downregulated in WT flies, including some that encode proteins with different abundances in *Wolbachia-*infected and uninfected mosquito cells [60]. Additionally, several putative cholesterol ester hydrolases responsible for making cholesterol and free fatty acids available to the cell when they are required for membrane and lipoprotein formation are downregulated in WT flies. Recent work on the human pathogenic bacterium *Chlamydia trachomatis* has demonstrated that cholesterol esterification is likely essential for cholesterol import into the membrane inclusion where the bacterium resides [61]. It is thus possible that the downregulation of genes that hydrolyze cholesterol esters in WT flies reflects a need for these molecules also by *Wolbachia*.

#### Reproduction

*Wolbachia* downregulates several genes involved in cell cycle, oocyte development, germ cell development and germ cell migration in OR female abdomens. Differential expression of these could potentially lead to phenotypes that are lethal for the embryo or which may cause defects in ovary development. An example of this is *Deadhead*, which is necessary for proper paternal chromatin decondensation during fertilization [62]. Interestingly, mutant maternal *Deadhead* can result in haploid embryonic development due to failed paternal chromatin condensation, a condition that resembles the CI phenotype induced by *Wolbachia* [32]. Further studies are still necessary to investigate whether CI occurs in *D. paulistorum*, but differential expression of such genes suggest *Wolbachia* might influence postmating compatibility in this species either through CI or other mechanisms.

#### Immunity

Our results show that *Wolbachia* influences the expression of genes associated with a wide range of immune responses in *D. paulistorum*. Among these, a clear pattern is seen on AMPs, which are consistently upregulated in WT flies, often with high fold change. Genes associated with melanization, opsonization, regulation of Toll and IMD pathways and control of nutrient availability are also affected, although with variable intensity and direction of regulation.

Different studies have linked symbionts in general and *Wolbachia* in particular to effects on the insect immune system. Tsetse flies, for example, become heavily immunocompromised if cleansed of their primary symbiont *Wigglesworthia* [63, 64], and mosquitoes develop increased resistance to viruses, bacteria, nematodes and protozoans when transinfected with *Wolbachia* [65]. Likewise, natural *Wolbachia* infections are known to provide protection against viruses and bacteria in *Drosophila* [66–68], although the mechanisms involved are not fully understood. Current hypotheses suggest the symbiont may directly or indirectly promote immune priming [65], activate the Toll and IMD pathways [69], or induce production of detoxifying agents and AMPs [70].

The upregulation of AMPs in WT *D. paulistorum* (Table 4, Additional files 3, 4 and 5) corroborates similar observations previously made in *D. melanogaster* and mosquito cell lines [49, 52, 53, 55]. Although this increase in AMP expression may be an infection-mediated immune response, it is also possible that the host needs to produce more of these molecules to control the number and localization of *Wolbachia* cells. In *D. paulistorum*, *Wolbachia* is localized in highly defined tissues and cell types such as the embryonic primordial germ cells [8], specific brain regions [17] and oenocytes [23]. Thus, one can hypothesize that AMPs could be used by the host to create and maintain this pattern in a similar way to what is observed in the weevil *Sitophilus zeamais,* which uses AMPs to restrict its bacterial endosymbiont to bacteriocytes. [71]. The fact that AMPs interact directly with their targets in a concentration dependent way could also explain why these molecules are generally DE with higher fold change than other immune genes, as larger changes in expression would be necessary for creating biologically relevant variations in their effect. Elevated levels of the AMP *Diptericin B* and of the immune gene *GNBP-like3* have also been recently correlated with enhanced long-term memory in *D. melanogaster* [72] suggesting that *Wolbachia*-mediated higher expression of AMPs in *D. paulistorum* might, directly or indirectly, improve host memory and thereby possibly affect sexual behavior [8]. It’s also worth noting the differential expression of genes associated with the Toll and IMD pathways, which mediate AMP production*.* Although only a few of the constituents of the pathway are affected, they might still have a relevant role in host immunity given their regulatory functions.

Finally, we also find several DE genes in OR associated with melanization, suggesting that the increase in this response induced by *w*MelPop in female mosquitos [73] could also be induced in *D. paulistorum* by its native *Wolbachia* infection.

#### Translation

The downregulation of ribosomal proteins and elongation factors in heads of OR WT flies suggests that *Wolbachia* suppresses host translation. Similar effects have been observed on the protein level in *Wolbachia*-infected *D. melanogaster* and *D. simulans* ovaries [74] and it is possible that they arise as a consequence of symbiont-mediated metabolic changes. Translation initiation is inhibited when the cell lacks essential amino acids such as leucine and methionine [75], hence, if severe enough, the appropriation of amino acids by *Wolbachia* could possibly reduce overall translation. However, it is unclear if such a lack of amino acids could result in a reduced expression of the ribosomal proteins and other genes involved in translation as observed here. Recent work suggests that *Wolbachia* titer increases if host translation is blocked [55], thus one possibility is that *Wolbachia* lowers translation in *D. paulistorum* heads in order to attain the high infection titer observed in the brain of this species [17].

### Novel functions affected by *Wolbachia*

#### Muscular functions

An unexpected number of genes related to muscular functions are upregulated by *Wolbachia* in both male and female abdomens. *Wolbachia* is known to infect muscles in *Drosophila* [18] and to increase locomotion in mosquitoes [76], but those observations are related to thoracic and not abdominal muscles, which is what we analyze here. One possibility is that *Wolbachia* might affect the heart, which in *Drosophila* is one of the largest skeletal muscles in the abdomen. Reduced expression of sarcomere genes has been connected to various cardiac diseases in *Drosophila* [77], and removing *Wolbachia* and thereby lowering the expression of such genes could possibly cause disease and lowered fitness in the flies. However, currently no phenotype connects *Wolbachia* and heart disease.

Although relatively little is known about the functions of abdominal muscles in *Drosophila* [78], one might speculate that altered muscle function could have implications in movement patterns associated with courtship and reproduction. The ventral abdominal muscles (VAMs), for example, are necessary for proper folding movements of the abdomen [78], and male *Drosophila* are known to use abdominal vibrations during courtship [79]. Hence, it is possible that changes in VAM activities could affect mating success. Another possibility is that the muscular genes observed to be DE in the abdominal muscles are also DE in thoracic muscles. If so, wing muscle function might be affected and have an impact on the generation of “love songs” by male flies. These songs are produced by rapid wing vibrations and have crucial role in *Drosophila* courtship by affecting female receptivity [79].

Finally, the large number of DE genes with muscle-related functions could also be a result of *Wolbachia*’s effect on host metabolism, since starvation induces a set of behavioral changes in *Drosophila* that enhances the search for food [80]. This behavioral change occurs through modulated perception of odors and tastes [81] as well as increased locomotor activity, which leads to a higher chance of finding food. One possibility is thus that the increased expression of muscle related genes might indicate that locomotion is increased in flies with WT levels of *Wolbachia*, possibly as a result of malnutrition. Again, we would have to assume that thoracic muscles are also affected, as much of the locomotion is supported by these muscle groups. Contrary to this hypothesis, though, the *adipokinetic hormone* (insect glucagon) *receptor* (*AkhR*) required for starvation-induced activity [80] has a significantly lower expression in WT flies, whereas the insulin-like receptor that was seen to counteract *AkhR*-induced locomotion, is instead upregulated. This is the opposite pattern of what would be expected if WT titers of *Wolbachia* lead to starvation-induced behavior.

Overall, an effect of *Wolbachia* on muscles, either directly or as a byproduct of metabolic changes, might impact courtship behavior and thus conceivably lead to the emergence of assortative mating.

#### Pheromone production and reception

Most DE genes involved in pheromone production participate in CHC synthesis and have a role in fatty acid metabolism. Among these, the fatty acid synthase *FASN2* has been implicated in the reproductive isolation between *D. serrata* and *D. birchii* [82]. In that case, selective pressure for different cuticle composition in populations living in contrasting humidity conditions probably led to divergence, as CHCs have a dual role as cuticle constituents and pheromones [82]. In an analogous way, one can hypothesize that *Wolbachia*-induced changes to fatty acid metabolism could affect the expression of *FASN* in *D. paulistorum*, leading to premating isolation between populations that respond differently to *Wolbachia* or which carry distinct symbiont strains. Importantly, *FASN2* is one of the few genes that are DE in all three semispecies (Fig. 2, Additional file 6). The influence of *Wolbachia* on *D. paulistorum* chemical communication is exemplified by a recent study showing that reduction of *Wolbachia* titer in males significantly affect semispecies-specific CHC profiles and triggers assortative mating of WT females against the symbiont-depleted mates [23].

Other fatty acid-related genes known to affect pheromone production in *Drosophila* are *desat1* and *desat2*, the latter of which is downregulated in CA and OR male WT abdomens. Desaturases create double bonds in CHC molecules, thus influencing the proportion of different compounds in the fly pheromone mix [26, 83]. *desat1* has been shown to affect sex pheromones of *D. melanogaster* [84], and both *desat1* and *2* are likely implicated in incipient speciation in the same species [83]*.*

Mechanisms for pheromone reception in *Drosophila* are generally poorly understood, but at least one OBP, *LUSH*, has been linked to responses to the courtship pheromone 11-cis-vaccenylacetate in *D. melanogaster* [85, 86]. Thus, the differential regulation of seven OBPs in OR flies can possibly affect pheromone response and consequently mate choice. Another protein involved in pheromone perception is the odorant degrading protein *Esterase 6*, here downregulated in heads of WT OR flies. It degrades odorants after they have bound to a receptor, thus allowing faster interaction with new molecules [41, 87].

Overall, the majority of the DE genes likely to be associated with pheromone production and reception are downregulated in OR WT heads and male abdomens, but a small number is upregulated in WT female abdomens. It is not clear why the direction of regulation in female abdomens is opposite to heads and male abdomens, but this pattern is seen in fatty acid metabolism genes, OBPs and Esterase 6, suggesting a biological reason might exist.

#### Vision

We found that several genes related to visual functions are upregulated in female heads of WT OR flies. *Wolbachia* is known to infect the optic lobe and the retina of *D. melanogaster* [18], and recent work in *D. paulistorum* showed that it also infects areas of the brain responsible for sensorial responses in that species, including vision [17]. It is not clear what biological consequences this has for the host, but one possibility is that it affects reproductive behavior, as vision has a documented importance in recognition of potential mates and perception of locomotor cues during *Drosophila* courtship [88, 89].

#### Yolk proteins

The *Wolbachia*-induced upregulation of yolk proteins in heads of WT *D. paulistorum* is rather intriguing given the usual association of these proteins with vitellogenesis. In female *D. melanogaster*, *Yp1-3* produce most of the components of egg yolk and are positively correlated with fertility, while in males they are implicated in sperm processing [90, 91]. Functions in the head are not known, although an association with the head fat body has been observed in *D. melanogaster* [92]. In the same species, yolk proteins are known to interact with the insect hormone ecdysone and to negatively impact longevity of both sexes [91]. So far, there is no clear connection between *Yp1-3* and any known *Wolbachia* phenotype, but it is interesting to note that the three yolk proteins are among the genes contributing the most to the separation between semispecies in the PCA of WT heads (Fig. 5).

### Does Wolbachia play a role in D. paulistorum speciation?

Mating between WT flies of different *D. paulistorum* semispecies has been shown to result in very low reproductive success, hybrid male sterility, and high rates of embryonic lethality [4, 8, 9, 11, 12]. In such conditions, it is expected that mechanisms would arise allowing individuals to recognize compatible mates before they waste energy and resources on unsuccessful reproductive attempts [93, 94]. *Wolbachia* most likely also benefits from preventing unfruitful host matings, as these are dead ends for a vertically transmitted symbiont. It seems plausible, then, that both host and symbiont would benefit if *Wolbachia* could enhance discrimination between *D. paulistorum* semispecies by inducing or enhancing some form of premating incompatibility.

Although our data doesn’t allow us to make definite conclusions regarding a role of *Wolbachia* in *D. paulistorum* speciation, especially in the case of low titer infections, several of our results support that hypothesis. Host premating isolation, for example, could be affected by DE genes involved in pheromone production and reception, as those might interfere with chemical communication. Genes associated to muscular functions might influence mating locomotor activities, including production of “love songs” through wing vibrations and abdominal tapping. Finally, genes which affect vision could impact recognition of mating cues and partner identification. The fact that genes affected by *Wolbachia* are overrepresented among those that contribute most towards distinguishing the gene expression between heads of AM, CA and OR flies (Fig. 5) also suggests that the symbiont might contribute to the emergence of behavioral differences between the semispecies, possibly including mate recognition.

Postmating isolation, on the other hand, could be influenced by many of the reproduction genes associated with cell cycle and germ cell development, as a disruption of their usual expression pattern could potentially harm or prevent embryonic development.

The strong metabolic changes observed in *D. paulistorum* as a result of *Wolbachia* infection also lead to the hypothesis that some or all of the functions with putative effects on speciation are a consequence of altered host metabolism. If correct, the metabolic cost of carrying *Wolbachia* might have causes physiological alterations which in turn impact reproductive behavior. Ultimately, those changes might have led to pre- and postmating isolation and speciation.

## Conclusions

The obligate relationship between *D. paulistorum* and *Wolbachia* combined with the ongoing divergence in the *D. paulistorum* complex results in a unique system for investigating symbiont-mediated speciation. Our results show that *Wolbachia* affects gene expression in different ways in two tissues and both sexes of three semispecies of *D. paulistorum*. The effect is particularly strong in OR, potentially due to the higher infection titer compared to the AM and CA semispecies.

Genes affected by *Wolbachia* are linked to a wide variety of biological functions. Some are globally responsive and previously known to be affected by the symbiont*,* such as immunity, reproduction and metabolism, while a few are novel tissue- or condition-specific functions, like those associated with muscles and vision. Our findings suggest that the competition between host and symbiont for amino acids, carbohydrates and lipids can be the cause of several of the physiological changes observed in *D. paulistorum* and that the association with *Wolbachia* either requires or leads to adjustments in the host immune functions.

We show that *Wolbachia* contributes to making the gene expression in heads more distinct between semispecies, supporting the hypothesis that the symbiont might influence mate choice and modulate host behavior. Furthermore, we suggest that a role for *Wolbachia* in the speciation of *D. paulistorum* is supported by the differential expression of genes involved in pheromone production and reception as well as reproduction and early embryonic development, as these are likely to influence pre- and postmating isolation between semispecies. It remains to be tested whether *Wolbachia* is a driving force of the speciation process or if it reinforces an already ongoing trend. In either case, we hypothesize that the possible contribution of *Wolbachia* to *D. paulistorum* semispecies isolation could be a benefit that maintains the infection in spite of the metabolic costs, as it might ultimately increase the chance of a fly identifying a suitable mate.

## Materials and methods

Flies were kept in Wolfgang Miller’s lab at the Medical University of Vienna and belong to three of the classical semispecies of *D. paulistorum*: Orinocan - line O11, Amazonian - line A28 and Centro American – line C2. These lines were obtained from Lee Ehrman, and descend from flies used in the experiments which helped define the classical *D. paulistorum* semispecies, back in the 1960s [5]. Flies were reared on Formula 4-24 ® instant food at 21-22 °C and 12 hour light/ dark cycle.

### Antibiotics treatment and gut flora restoration

In order to knock down the *Wolbachia* infection, WT flies were kept on food containing Rifampicin 0.2% w/v for three consecutive generations according to [8]. PCR screenings targeting the *Wolbachia wsp* gene showed that infection titer was reduced to below detection level after treatment. Gut flora restoration was done by transferring treated flies to tubes containing regular food in which virgin WT females of the corresponding semispecies had been kept for 2-3 days, so that feces had accumulated on the food and inner surfaces. After egg deposition, adults were removed and the larvae which developed in those vials were considered gut flora restored.

### Sample collection and RNA extraction

Whole heads, including brain and mouthparts, and abdomens, containing both gonads and gut, were severed from 3-day old adult females and males of either WT or F3 generation GFR flies using fine tweezers. No attempt was made to keep flies virgins. A total of approximately 20 heads and 10 abdomens were pooled per head and abdomen sample, respectively. Three biological samples were collected per condition and RNA was subsequently extracted using a TRIzol™ Reagent protocol (Sigma). In brief, samples were homogenized in TRIzol™ reagent with sterile pestles, incubated at room temperature, and phases were separated using 1–bromo–3–chloropropane (BCP) phase separation reagent. RNA was collected from the aqueous phase, transferred into new tubes and precipitated with isopropanol. Pellets were washed, dried and rehydrated in RNase free water.

### Sequencing and read quality control

The extracted RNA was used for library preparation at the SNP&SEQ Technology Platform in Uppsala, Sweden. Per sample, a total of 0.5 μg of RNA was treated with the ScriptSeq complete Gold Epidemiology kit (Illumina, part# BEP1224) for rRNA depletion according to the manufacturer’s protocol (Lit#356-4-2013 RevA). The kit was originally designed for human, mouse, rat and bacterial samples, but successful use in *Drosophila* is reported at the manufacturer’s species compatibility table. Sequencing was done with Illumina HiSeq2500, to produce paired-end 125 bp reads using v4 sequencing chemistry. All 72 samples were run in the same Illumina flowcell. Samples from the different conditions (sex, treatment, semispecies) were arranged so that replicates of the same condition were run in different lanes and with molecular identifiers rotated between samples to avoid any systematic bias.

Sequenced reads were quality-checked with FastQC v0.10.0 [95] and processed with Trimmomatic v0.36 [96] for residual adapter removal and mild quality trimming using the parameters: ILLUMINACLIP:TruSeq3-PE.fa:2:40:15 LEADING:3 TRAILING:3 SLIDINGWINDOW:4:15 MINLEN:95

### Transcriptome assembly and ORF prediction

Transcriptomes of each semispecies were separately assembled with Trinity v2.1.1 [97] using the parameters “--min_kmer_cov 2” and “--normalize_reads”. All samples of each semispecies were used for the respective assembly with the exception of O11-GFR-F-abd3 and O11-WT-M-abd3, for the OR assembly, and C2-WT-abd1, for CA assembly, which contained higher than average rRNA level as indicated by the reports from the sequencing facility. Contigs within one Trinity gene group are referred to as “genes” as they likely represent isoforms or possible assembly artefacts from the same gene.

Transcriptome completeness was evaluated with BUSCO v3.0.2 [98] using Arthropoda, Insecta and Diptera markers. Assembly quality was further assessed through the percentage of reads mapping back to their respective assembly using BWA mem aligner [99] and by calculating N50, average contig length and percentage of complete ORFs.

An estimate of the percentage of reads mapping to different organisms was obtained for each assembly through competitive mapping with BWA mem to a reference including genomes of *Drosophila*, *Wolbachia*, yeast and *Drosophila* gut bacteria (Additional file 1).

Open reading frames were predicted with TransDecoder v2.0.1 [100] using the “-S” flag for paired end reads. Identified ORFs were aligned to PFAM (Release 31.0) with HMMer v3.1b2 [101] and to Swissprot (Release 2017_10) with BLAST v2.2.20. The resulting matches were used as input to TransDecoder together with the previously detected ORFs for a refined predictive round using the flags “--retain_pfam_hits” and “–retain_blastp_hits”.

### Differential expression analysis

In each assembly, contigs containing multiple ORFs were split and replaced by the corresponding ORFs. The resulting sequences were then clustered with CD-HIT-est [102] using a 100% identity cutoff. This procedure removed redundancy while preserving as much as possible the assembled sequence diversity so as to reduce the risk of inducing misalignments during mapping. Reads from each semispecies were mapped to its corresponding reference using STAR v2.5.2b [103] with default parameters. Some of the mapping statistics produced by STAR are presented in Additional file 1, including the percentage of reads mapping to the assemblies and the percentage of mismatches per base.

Reads mapping to each contig were counted with FeatureCounts [104] using the flags “-M”, “-s 1” and “-p” for counting multimapping reads, taking strand information into account, and counting fragments instead of reads, respectively. Contigs in the subsequent count table were clustered using CD-HIT with 98% identity cutoff at amino acid level for decreasing redundancy, removal of non-coding RNAs and reducing downstream issues with multiple testing. The resulting contigs and respective read counts were used as reference for the differential expression analysis. Contigs were not removed from the transcriptomes with basis on which organism they were associated with (*Drosophila*, *Wolbachia*, yeast or other bacteria) so as to avoid misalignments during mapping.

The differential expression analysis was done in R v3.2.2 [105] with the DESeq2 v2_1.10.1 [106] package, which uses an inbuilt normalization pipeline. Tests were performed between WT and GFR samples of heads or abdomens of each sex. GFR was set as reference condition so that difference in expression could be read as being induced by *Wolbachia*. Contigs were considered differentially expressed if an adjusted pvalue (qvalue) ≤ 0.05 was observed in DESeq2’s default Wald test. Contigs were said to be differentially expressed with high fold change whenever their absolute fold change value was greater than one standard deviation from the mean absolute fold change for the condition in which they were DE.

### Plots and statistics

Principal component analyses were performed with DESeq2 and plotted with ggplot2 [107]. PCAs of individual semispecies were based on reads of that semispecies mapped to its own reference transcriptome, while PCAs of multiple semispecies are based on reads of all semispecies mapped to the OR reference transcriptome. Genes which contributed the most to each principal component (PC) were identified by their loading values. These were obtained by extracting the “rotation” element when calculating the PCA using the prcomp() function in R. Loadings were then plotted in ascending order and the genes whose values stood out in the beginning or end of the curve were selected. A chi-squared test was used to verify whether DE genes between GFR and WT flies were significantly overrepresented (pvalue < 0.05) among the genes which contributed the most to the separation between semispecies in the PCA plots.

Heatmaps of individual semispecies were generated with ggplot2 and were based on the expression values obtained in DESeq2 for the contigs DE between WT and GFR for that semispecies.

Venn diagrams were created in R using the VennDiagrams package [108] and are based on *Drosophila* genes identified as homologous between semispecies after clustering of the three differential expression reference transcriptomes with OrthoMCL [109] using default parameters. Venn diagram in Fig. 2 shows only *Drosophila* DE genes while the one in Additional file 2: Figure S1 includes all *Drosophila* genes — whether DE or not. BLAST searches to identify organisms associated to the genes associated to a single transcriptome in Additional file 2: Figure S1 were performed with protein BLAST against the non-redundant database of NCBI. Plots showing the number of DE genes in one or in multiple conditions were made in R with the upsetR package [110].

### Contig and gene annotation

DE contigs were annotated using two independent strategies. In the first one, all contigs were run through Interproscan v5.24-63.0 for GO term annotation.

In the second strategy, all contigs were blasted to a database containing genes from *Drosophila willistoni*, *Drosophila melanogaster*, *Wolbachia*, the yeast *Saccharomyces cerevisiae* and a number of *Drosophila* gut bacteria (Additional file 1). Contigs with a best hit to *Wolbachia*, other bacteria or yeast were discarded, while those with higher similarity to *Drosophila* were considered for annotation if the following criteria were met: the length of either the query or the subject, whichever was shortest, should correspond to at least 60% of the length of the longest, and the size of the aligned segment should correspond to at least 80% of the length of the shortest sequence.

Among the contigs which fulfilled these criteria, those with a best hit to *D. melanogaster* were directly annotated according to the Flybase *D. melanogaster* gff annotation file (release 6.18), while those with a best hit to *D. willistoni* had their annotation inferred from *D. melanogaster* orthologs listed in the Flybase gene ortholog table v2017_04. GO terms for each annotated contigs were extracted from the Flybase go-basic.obo file (release 2017-04-19). GO terms obtained through this method were generally considered more detailed than the ones annotated through InterproScan and were thus used for GO term enrichment analysis with the R package TopGo v2.22.0 [111]. TopGO’s default “weight01” algorithm and Fisher’s exact test statistic were employed in the analysis and GO terms were considered enriched when an adjusted pvalue < 0.05 was obtained. In order to avoid biasing the GO enrichment analysis with eventual multiple copies of a same transcript, the analysis was performed on a dataset containing only one contig for each assembled “Trinity gene”. This dataset was created using the script “extract_GO_assignments_from_Trinotate_xls.pl”, available with the Trinotate software package [112], which annotates each “Trinity gene” with the GO terms of all the contigs associated with it.

Contigs identified as differentially expressed were mapped against the KEGG (http://www.kegg.jp/) database for identification of metabolic pathways associated with them. The online tool blastKOALA (http://www.kegg.jp/blastkoala/) was used for this purposed, with Taxonomy ID set to 7215 (*Drosophila*) and KEGG gene database set to “family_eukaryotes + genus_prokaryotes”. The metabolic map in Fig. 4 was prepared with the “search&color pathway” function of the KEGG mapper tool (https://www.genome.jp/kegg/mapper.html) and redrawn in Adobe Illustrator.

## Additional files

Additional file 1: Assembly metrics for the mapping reference transcriptomes of the AM, CA and OR semispecies. Transcriptome assembly metrics, including BUSCO marker recovery, ORF prediction and completeness, and the percentage of reads and ORFs associated with different organisms. (XLSX 24 kb)

Additional file 2: Additional figures. **Figure S1.** Overlap in *Drosophila* gene content between the transcriptomes of the three semispecies. **Figure S2.** Principal component analysis (a) and heatmap (b) of expression data of the AM semispecies. **Figure S3.** Principal component analysis (a) and heatmap (b) of expression data of the CA semispecies. **Figure S4.** Number of genes differentially expressed in one or multiple conditions of the AM semispecies. **Figure S5.** Number of genes differentially expressed in one or multiple conditions of the CA semispecies. **Figure S6.** First and second principal components in the PCA of female abdomen samples of all semispecies. **Figure S7.** Second and third principal components in the PCA of head samples of all semispecies mapped to the OR transcriptome. **Figure S8.** Principal component analysis of GFR head samples of all semispecies mapped to the OR transcriptome based on the same genes used in the WT head PCAs (Figs. 5c, 2d). **Figure S9.** Principal component analysis of abdomen samples of all semispecies mapped to the OR transcriptome. (PDF 2115 kb)

Additional file 3: All DE genes in the OR semispecies. Complete list of all DE genes in the OR semispecies including which condition, sex and tissue it was DE in, fold change, significance value, annotated Flybase gene identities for orthologs in *D. willistoni* and *D. melanogaster*, annotated gene name and domain predictions. (XLSX 505 kb)

Additional file 4: All DE genes in the CA semispecies. Complete list of all DE genes in the CA semispecies including which condition, sex and tissue it was DE in, fold change, significance value, annotated Flybase gene identities for orthologs in *D. willistoni* and *D. melanogaster*, annotated gene name and domain predictions. (XLSX 67 kb)

Additional file 5: All DE genes in the AM semispecies. Complete list of all DE genes in the AM semispecies including which condition, sex and tissue it was DE in, fold change, significance value, annotated Flybase gene identities for orthologs in *D. willistoni* and *D. melanogaster*, annotated gene name and domain predictions. (XLSX 69 kb)

Additional file 6: DE genes in multiple semispecies. List of all DE genes in more than one semispecies. (XLSX 17 kb)

Additional file 7: All enriched GO terms in the AM, CA and OR semispecies. List of all GO terms enriched in each condition of the three semispecies. (XLSX 48 kb)

## Data Availability

The datasets generated and analyzed during the current study are available in the SRA database at NCBI, accession number PRJNA515416, https://www.ncbi.nlm.nih.gov/bioproject/PRJNA515416

## Abbreviations

AB: Andean-Brazilian
AM: Amazonian
AMP: Antimicrobial peptide
BCP: 1–bromo–3–chloropropane
CA: Centro-American
CHC: Cuticle hydrocarbon
CI: Cytoplasmic incompatibility
DE: Differentially expressed
GFR: Gut flora restored
GO: Gene ontology
IN: Interior
OBP: Odorant-binding protein
OR: Orinocan
ORF: Open reading frame
PC: Principal component
PCA: Principal component analysis
THF: Tetrahydrofolate
TR: Transitional
VAM: Ventral abdominal muscle
WT: Wild type
