# Supplementary material for: The effect of Wolbachia on gene expression in Drosophila paulistorum and its implications for symbiont-induced host speciation
Source: BMC Genomics. 2019 Jun 7;20:465. doi: 10.1186/s12864-019-5816-9 (PMC6555960; doi:10.1186/s12864-019-5816-9)
Supplement: Supplementary file 2 — Additional figures. Figure S1. Overlap in Drosophila gene content between the transcriptomes of the three semispecies. Figure S2. Principal component analysis (a) and heatmap (b) of expression data of the AM semispecies. Figure S3. Principal component analysis (a) and heatmap (b) of expression data of the CA semispecies. Figure S4. Number of genes differentially expressed in one or multiple conditions of the AM semispecies. Figure S5. Number of genes differentially expressed in one or multiple conditions of the CA semispecies. Figure S6. First and second principal components in the PCA of female abdomen samples of all semispecies. Figure S7. Second and third principal components in the PCA of head samples of all semispecies mapped to the OR transcriptome. Figure S8. Principal component analysis of GFR head samples of all semispecies mapped to the OR transcriptome based on the same genes used in the WT head PCAs (Figs. 5c, 2d). Figure S9. Principal component analysis of abdomen samples of all semispecies mapped to the OR transcriptome. (PDF 2115 kb) [file 12864_2019_5816_MOESM2_ESM.pdf]

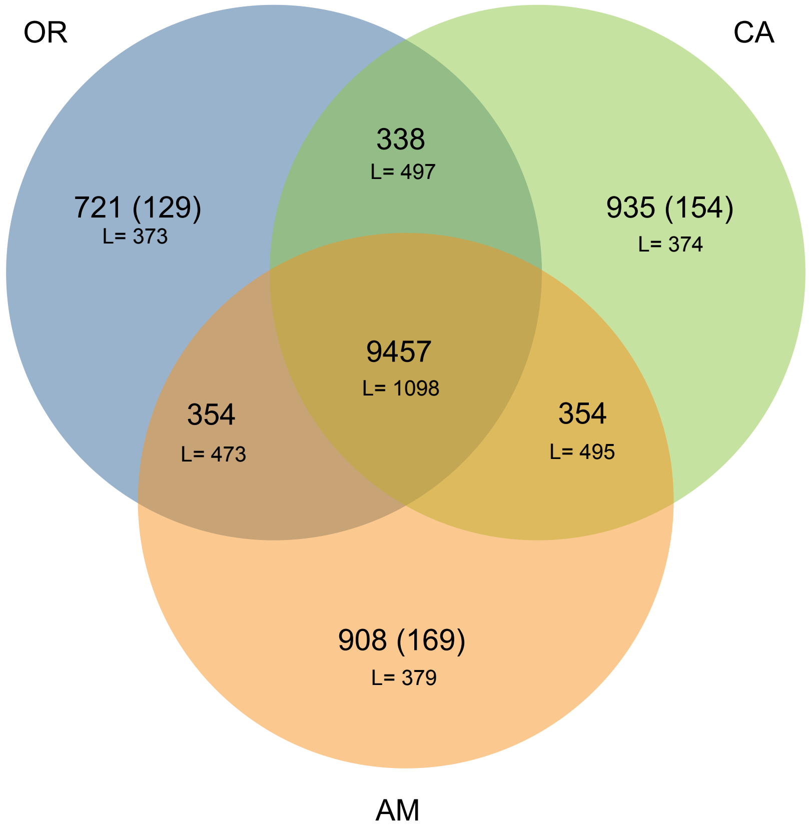

Figure S1: Overlap in gene content between the transcriptomes of the three semispecies. Numbers represent clusters obtained by running OrthoMCL on the DE reference transcriptomes of the three semispecies. Contigs associated with bacteria, yeast and *Wolbachia* were removed before clustering. The 9457 clusters shared the three semispecies include 30577, 30404 and 30049 contigs from AM, CA and OR, respectively. These correspond to the vast majority of *Drosophila* contigs in each semispecies. Most genes identified as unique to a semispecies probably represent misassemblies or fragments, as they couldn't be assigned to any organism and have much smaller length than those which are shared. Numbers in brackets are genes unique to a semispecies which could be assigned to *Drosophila*. Only a very small number of unique genes are DE — 4, 2 and 6 for AM, CA and OR, respectively — none of which could be annotated. L: Average length of contigs.

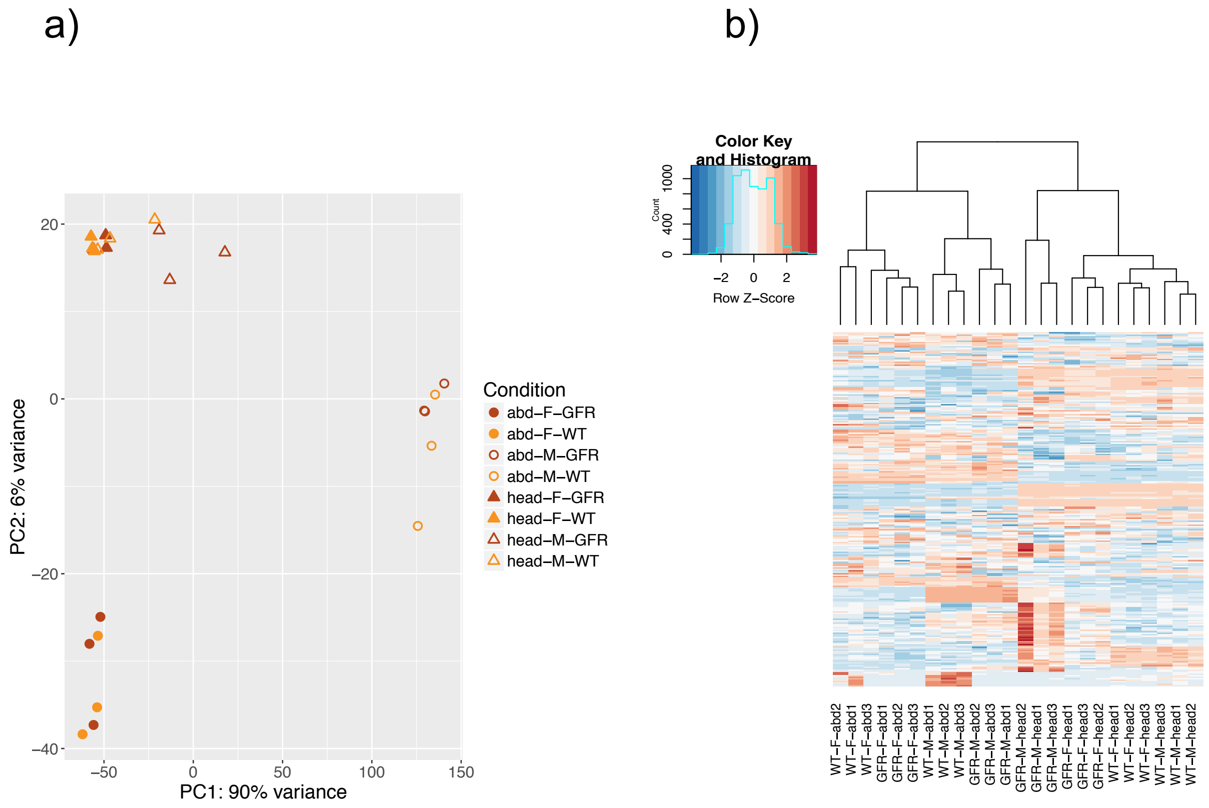

Figure S2: Principal Component Analysis (a) and heatmap (b) of expression data of the AM semispecies. The PCA is based on all *Drosophila* genes included in the analysis, while the heatmap shows only DE genes. F: female, M: male, WT: wild type, GFR: gut flora restored, abd: abdomen

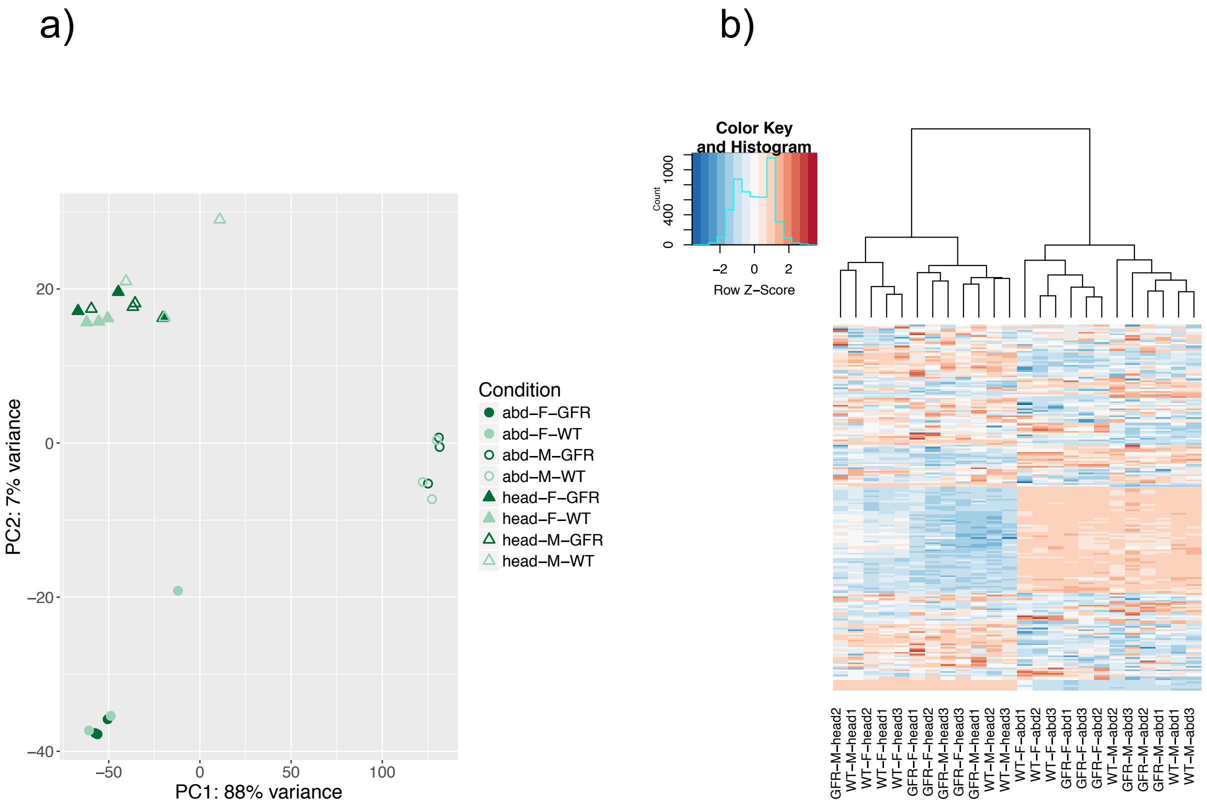

Figure S3: Principal Component Analysis (a) and heatmap (b) of expression data of the CA semispecies. The PCA is based on all *Drosophila* genes included in the analysis, while the heatmap shows only DE genes. F: female, M: male, WT: wild type, GFR: gut flora restored, abd: abdomen

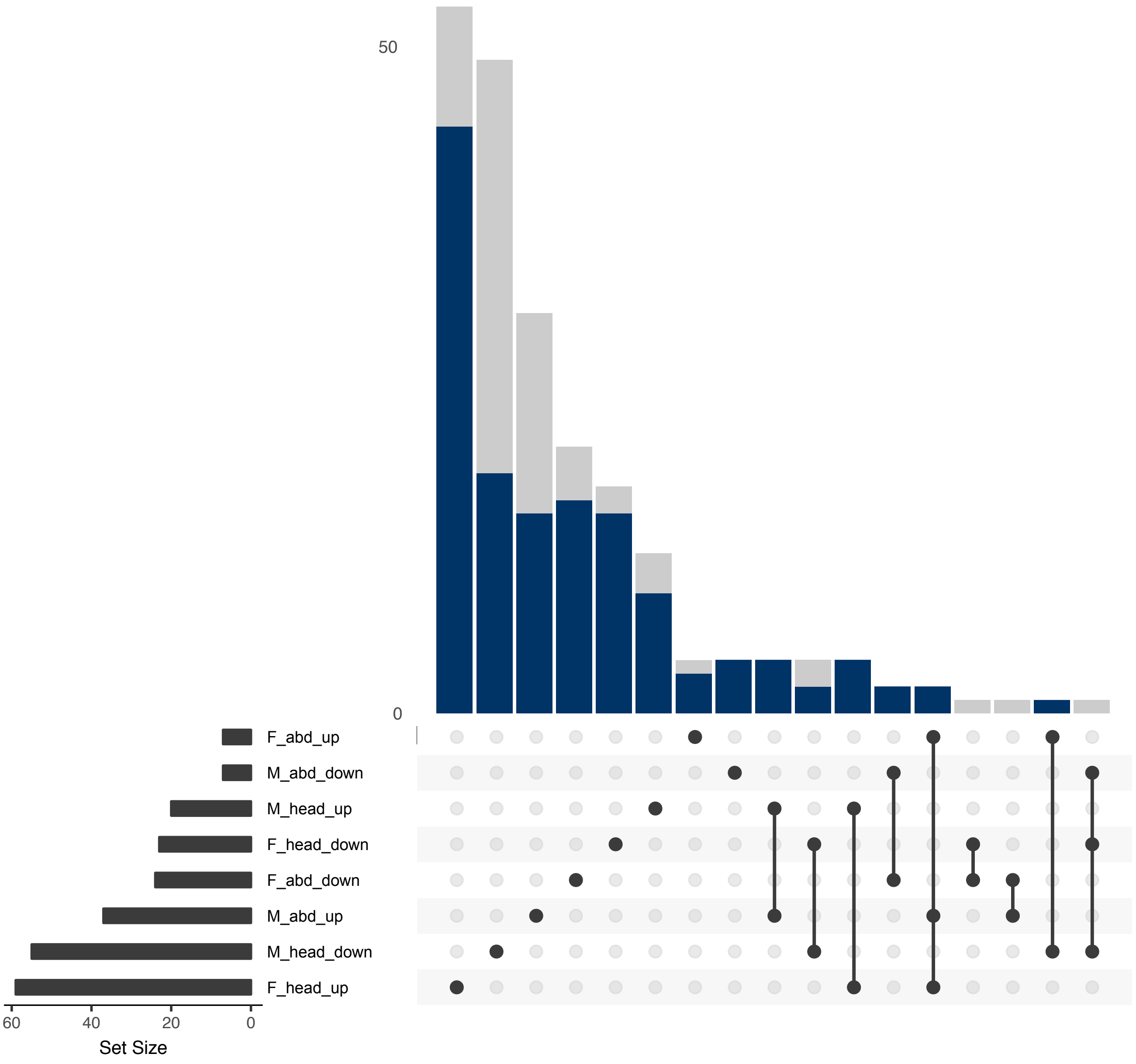

Figure S4: Number of genes differentially expressed in one or multiple conditions of the AM semispecies. A black dot indicates the presence of DE genes for the condition named on the left side and dots linked by lines represent DE genes in multiple conditions. Vertical bars above the dots correspond to the number of annotated (blue) and unannotated (grey) DE genes present in the condition(s) with a dot. Horizontal black bars on the lower left indicate how many genes are DE in each condition. F: female, M: male, WT: wild type, GFR: gut flora restored, abd: abdomen, up: upregulated, down: downregulated

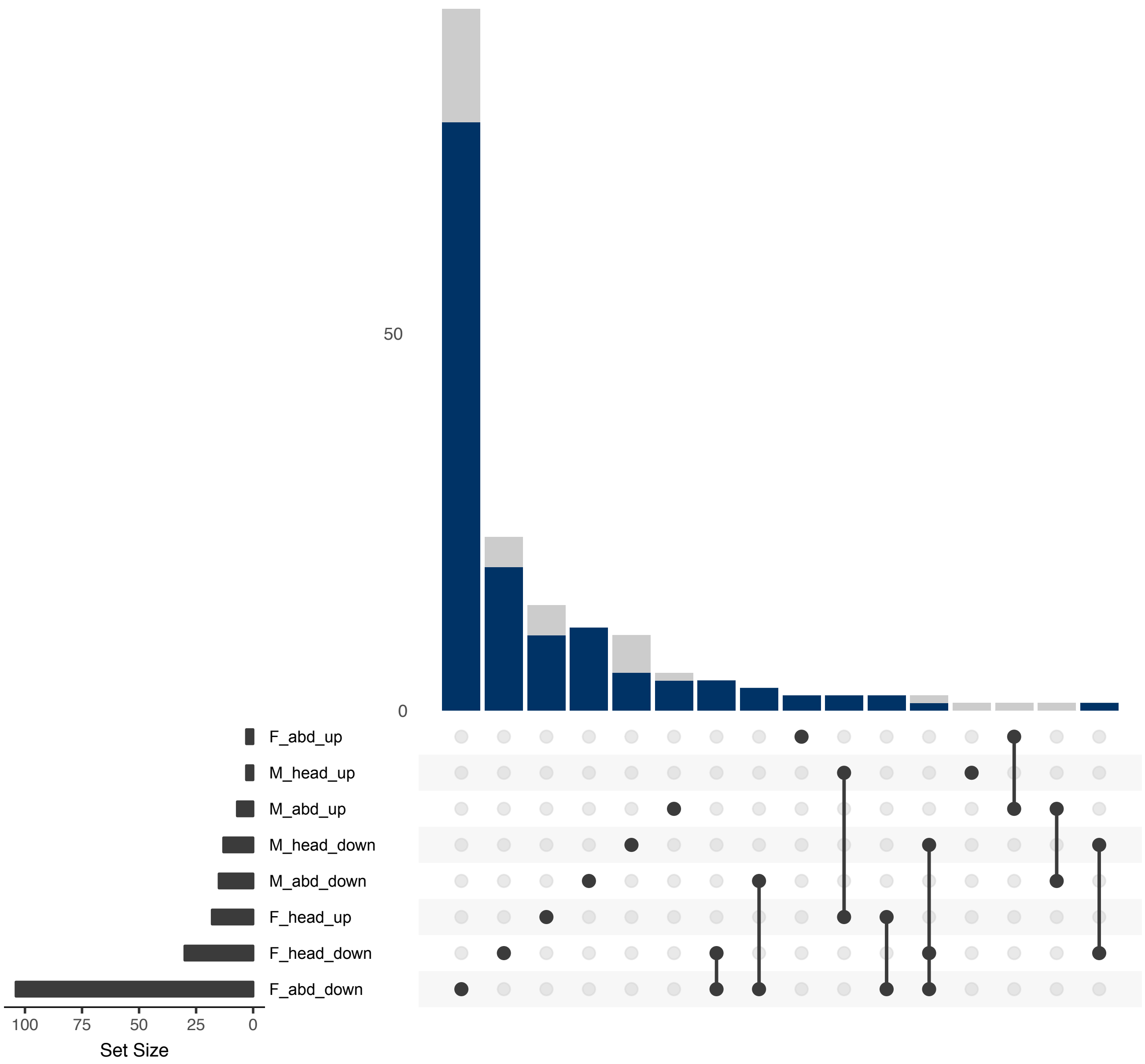

Figure S5: Number of genes differentially expressed in one or multiple conditions of the CA semispecies. A black dot indicates the presence of DE genes for the condition named on the left side and dots linked by lines represent DE genes in multiple conditions. Vertical bars above the dots correspond to the number of annotated (blue) and unannotated (grey) DE genes present in the condition(s) with a dot. Horizontal black bars on the lower left indicate how many genes are DE in each condition. F: female, M: male, WT: wild type, GFR: gut flora restored, abd: abdomen, up: upregulated, down: downregulated.

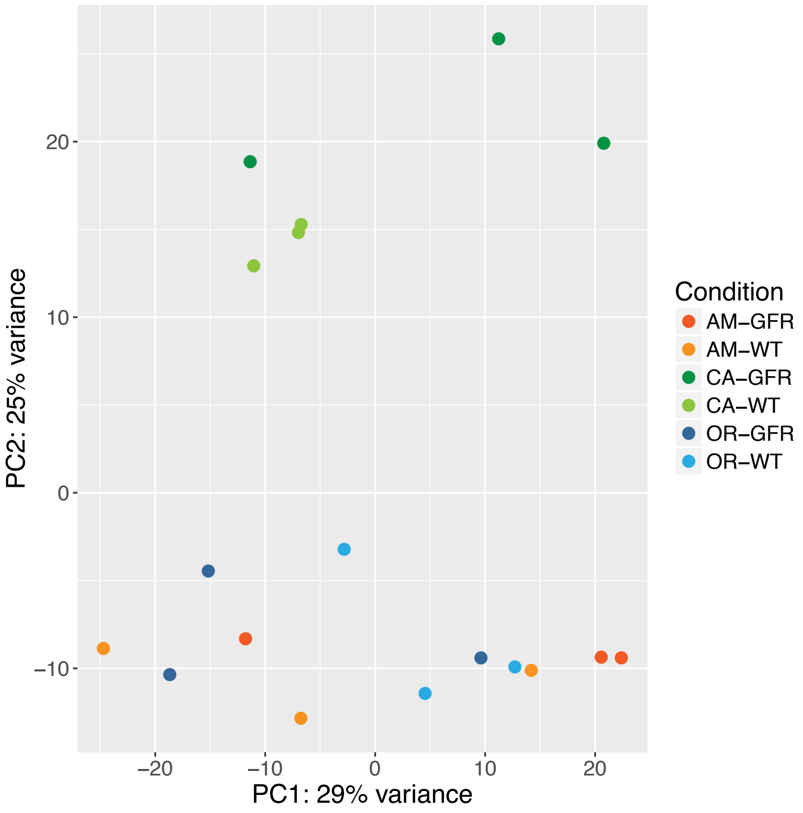

Figure S6: First and second principal components in the PCA of female abdomen samples of all semispecies. Plot complementary to Figure 2a. WT: wild type. GFR: Gut flora restored

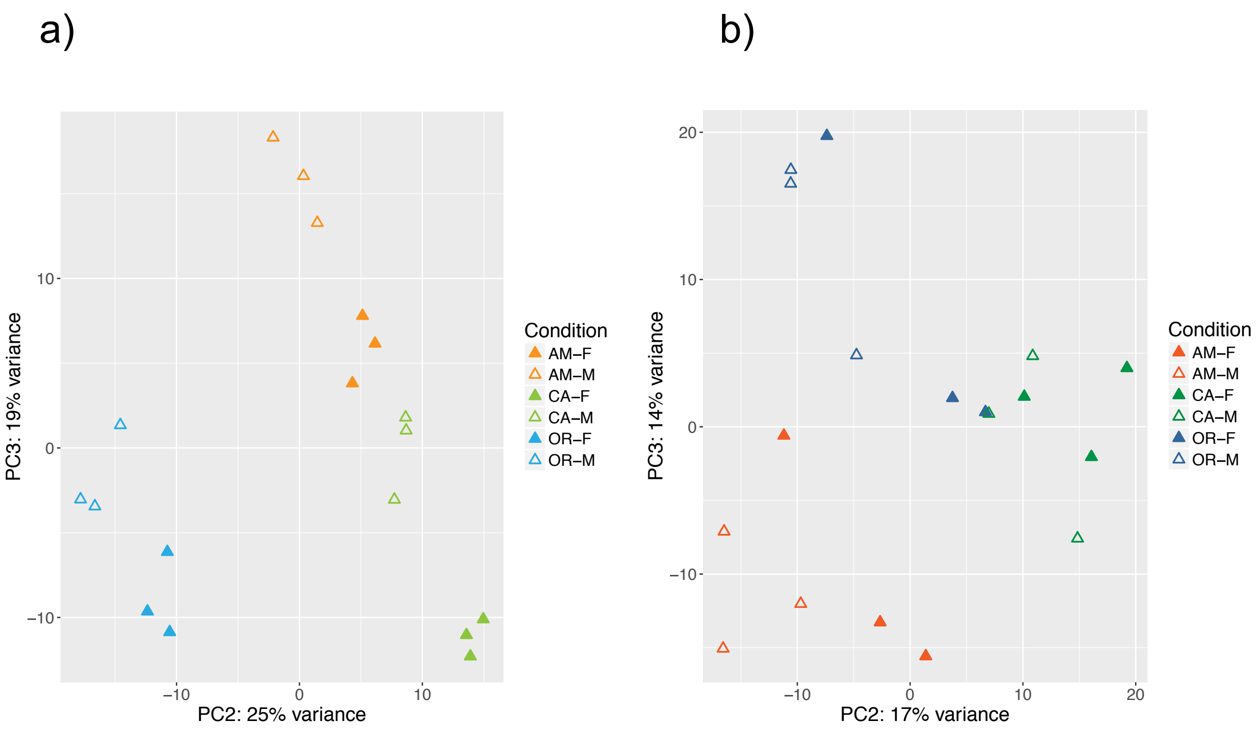

Figure S7: Second and third principal components in the PCA of head samples of all semispecies mapped to the OR transcriptome. Plots complementary to Figures 2c, 2d. (a): WT heads, PC2 x PC3 (b): GFR heads, PC2 x PC 3. F: female, M: male

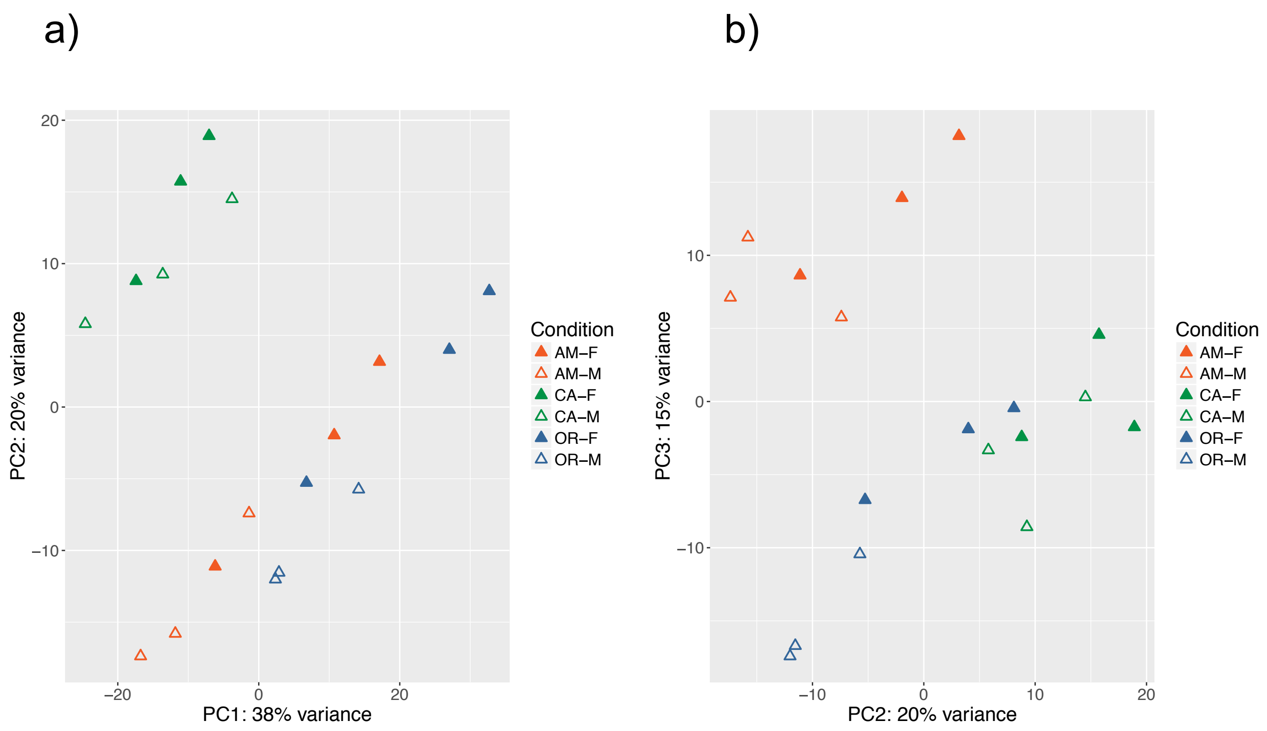

Figure S8: Principal component analysis of GFR head samples of all semispecies mapped to the OR transcriptome, based on the same genes used in the WT head PCAs (Figure 2c, 2d). (a): PC1 x PC2, (b): PC2 x PC 3. F: female, M: male

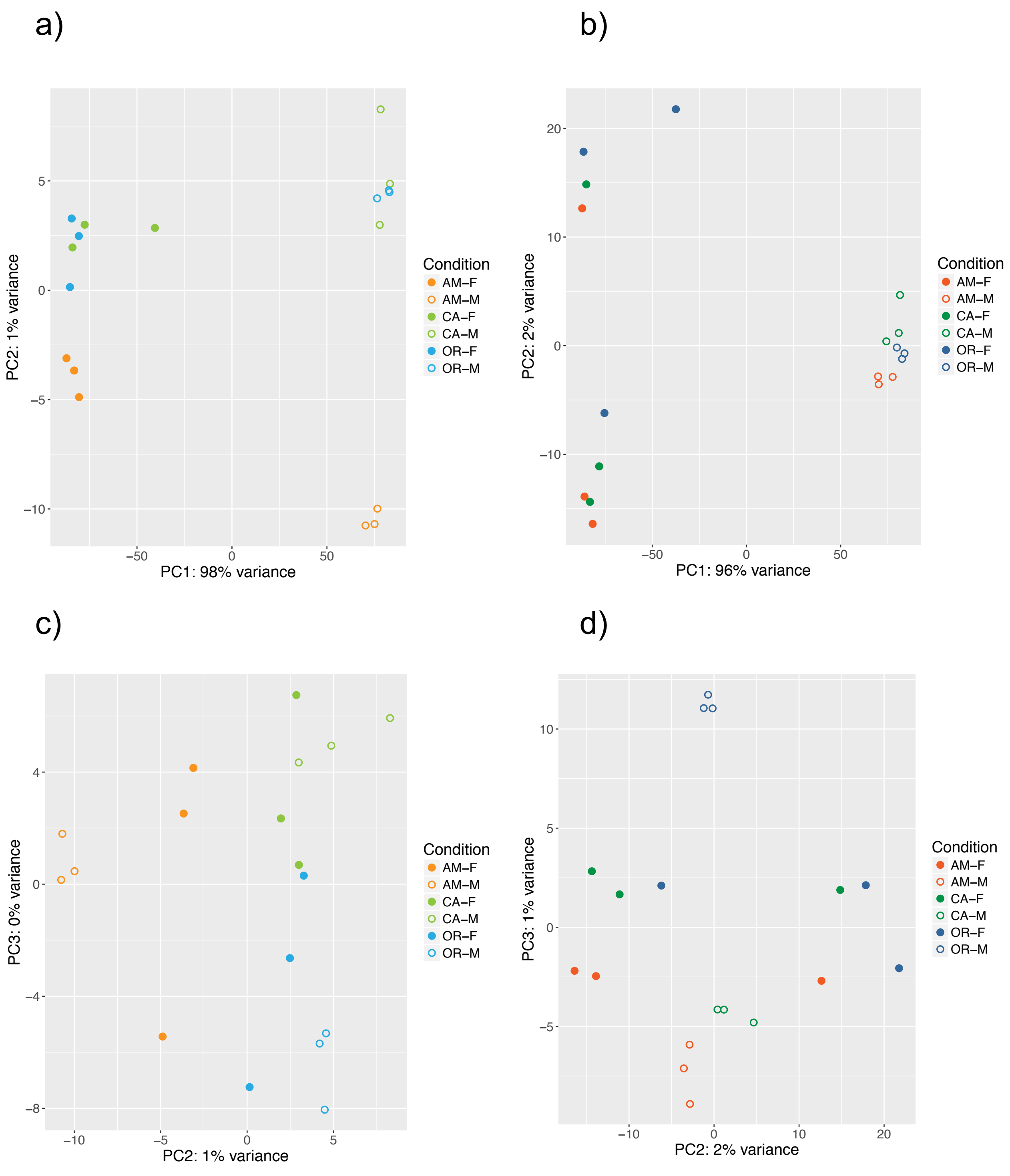

Figure S9: Principal component analysis of abdomen samples of all semispecies mapped to the OR transcriptome. (a): WT abdomens, PC1 x PC2 (b): GFR abdomens, PC1 x PC2 c) WT abdomens PC2 x PC3 d) GFR abdomens, PC2 x PC3. F: female, M: male
